# Mechanistic insight into the dynamics of Mur ligase through a comprehensive timescale-specific approach

**DOI:** 10.1038/s42004-025-01675-z

**Published:** 2025-09-29

**Authors:** Iza Ogris, Barbara Zupančič, Izidor Sosič, Franci Merzel, Simona Golič Grdadolnik

**Affiliations:** 1 Laboratory for Molecular Structural Dynamics, Theory Department, National Institute of Chemistry, Ljubljana, Slovenia; 2 Faculty of Medicine, University of Ljubljana, Ljubljana, Slovenia; 3 Faculty of Pharmacy, University of Ljubljana, Ljubljana, Slovenia

**Keywords:** Solution-state NMR, Ligases

## Abstract

Muramyl ligases are multidomain enzymes involved in intracellular steps of bacterial peptidoglycan synthesis and are considered promising targets for the development of new antibacterial agents. Among them, Mur ligase D (MurD) has been most widely used for structure-based design, but success has been limited. Here, we determine the ^15^N NMR spin relaxation parameters of apo and bound states of MurD in solution. We introduce a principal component analysis of the spectral densities derived from the NMR relaxation data, which provides a mechanistic insight into the dynamic events at the residue level. Compensation effects (ps-ms timescale) and conformational exchange dynamics (µs-ms timescale), the latter also measured independently, were revealed in bound and unbound MurD, which should be considered in the design of structurally novel Mur inhibitors. The mechanistic consideration used in our study can be broadly applicable to other systems for deciphering their specific dynamic mechanisms.

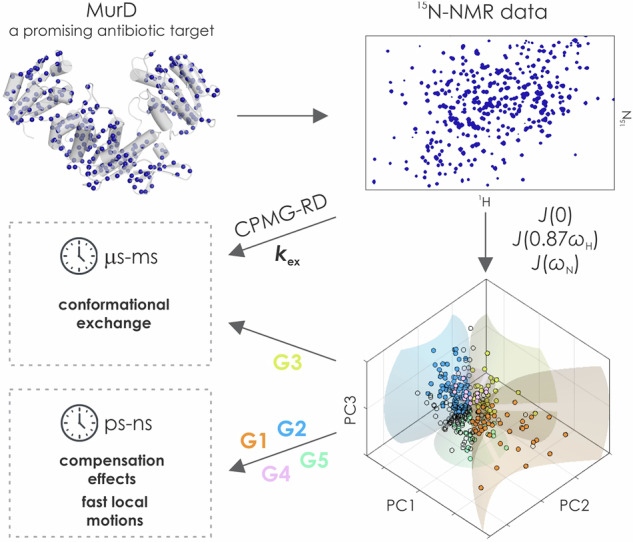

## Abstract (web summary)

Mur ligase D (MurD) is a promising antibacterial target, but its dynamics remain underexplored. Here, the authors use ^15^N nuclear magnetic resonance to probe apo and bound states of MurD in solution, showing that dynamic events on a time scale of picoseconds to milliseconds have to be considered in the design of structurally novel Mur inhibitors.

## Introduction

The Mur ligase superfamily, encompassing MurC, MurD, MurE and MurF, plays a pivotal role in intracellular peptidoglycan synthesis, thereby attracting considerable attention as potential targets for the development of novel antibacterial agents^1^. These ATP-dependent ligases demonstrate structural dynamics that are intrinsically linked to their capacity to bind to and catalyze the formation of considerably large peptidoglycan precursors. As the subsequent addition of amino acids to the precursors progresses from MurC to MurF, it enables the generation of increasingly larger building blocks for peptidoglycan synthesis. Consequently, the conformational dynamics among these four ligases exhibit a notable increase, reflecting their respective roles in the elongation of the peptidoglycan chain^2–5^.

Among the Mur ligases, MurD (UDP-*N*-acetylmuramoyl-l-alanyl-d-glutamate ligase) - a 47.7 kDa multidomain protein - is the most extensively studied target for inhibitor design^6–20^. Based on crystallographic data, it has been observed that MurD can adopt several distinct conformational states during the catalytic process, including:

- An open conformation, representing the ligand-free conformation of MurD in the absence of a ligand^21^.
- A semi-closed conformation, initially adopted upon ATP and Mg²⁺ binding to central domain (CeD, residues 94-298) at the interface with C-terminal domain (CTD, residues 299-437), which follows the binding of the substrate UDP-*N*-acetylmuramoyl-l-alanine (UMA) to the cleft formed between the N-terminal domain (NTD, residues 1-93) and CeD^22,23^.
- A closed conformation, which is adopted after ATP hydrolysis, and is further characterized by d-Glu binding to CTD and the subsequent formation of the product UMAG^24,25^. Furthermore, the closed conformation is adopted after binding some of its most potent inhibitors, transition state analogs^9–11,14,16,26^, indicating that this conformation could be crucial for regulating MurD activity.

Notably, the rigid body rotation of the CTD facilitates transitions between open, semi-closed to closed conformation, as evident from crystallographic studies^16,21,24,25^ and shown with pseudocontact shift (PCS) NMR studies, which also reveals that this motion regulates substrate binding affinity^27^.

The concept of semi-closed conformation was first introduced through PCS-NMR studies^27^. Later, two crystal structures called intermediate-apo (PDB 5A5E) and intermediate-UMA + ADP bound (PDB 5A5F) conformation were solved^23^. MD simulations showed that both, together with the PCS-model, lie within the conformational ensemble of the simulated ATP-bound state, suggesting that the 5A5E and 5A5F crystal structures represent the semi-closed conformation^28^. The recent ATP-bound crystal structure (PDB 8VW2)^22^ also closely resembles these crystal structures based on RMSD comparisons (Table S1).

Recently, electron paramagnetic resonance (EPR) of frozen solutions^29^ and small-angle X-ray scattering (SAXS) experiments in solution^30^ have identified a wide range of distances between the CTD and the CeD, revealing the existence of multiple conformational states in the apo MurD. In the most populated state, this distance was consistent with the distance observed in the crystal structure of the open conformation. However, in the conformational ensemble, a state was also found in which the distance corresponds to the closed conformation. Upon inhibitor binding, the distance distribution narrowed and the conformational ensemble shifted towards the distance closely resembling those in the closed conformation observed in the crystal structures^29,30^. This supports the idea that conformational selection is a key mechanism in ligand binding.

While efforts to develop potent MurD inhibitors have achieved some success, the efficacy has been somewhat limited, with IC_50_ values reaching only into the single-digit micromolar (µM) range^11,26,31^. Interestingly, the campaigns to find competitive ATP inhibitors^6,12,18^ that could lead to multi-target inhibitors of the entire Mur ligase series yielded inhibitors that bind to UMA or d-Glu instead to the ATP binding site. Since all these efforts were based on the rigid crystallographic structures, we can speculate that the lack of knowledge about the dynamic properties of the apo and bound MurD states has hindered further development that could eventually lead to structurally novel antibacterial agents with clinical potential.

Here, by using ^15^N nuclear magnetic resonance (NMR) relaxation techniques in solution accompanied by a specific analysis of NMR data and supported by molecular dynamics (MD) simulations we unravel a complex dynamic behavior of three MurD states: apo MurD (MurD_apo_), MurD bound with stable ATP analog AMP-PCP (MurD_ACP_), and MurD bound with the most potent sulfonamide type inhibitor (4-(6-(4-cyano-2-fluorobenzyloxy)naphtalene-2-sulfonamido)isophthalic acid, inhibitor **1**) hereafter indicated as MurD_inh_. Reduced Spectral Density Mapping (RSDM) was employed to identify the distinct characteristics of the reduced spectral densities *J*(*ω*) along the protein backbone in the three states that depict the residues and regions with varying dynamics between the states from ps to ms time scale. By introducing Principal Component Analysis (PCA) into the spectral density data, we revealed three independent dynamic mechanisms corresponding to different intertwining of the time scales of the dynamic events that was not possible with usually applied pairwise comparison of *J*-terms. We have shown that an in-depth characterization of the dynamic mechanisms of apo and bound MurD forms is of paramount importance for a deeper understanding of the binding mechanism of substrates and inhibitors and consequently for the successful development of effective and more potent inhibitors.

## Results

### Determination of spectral densities from ^15^N NMR relaxation data

**Fig. 1:**
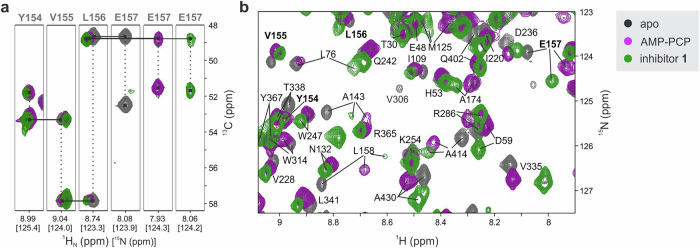
Expanded regions of the NMR spectra of MurD_apo_ (in gray), MurD_ACP_ (in magenta) and MurD_inh_ (in green), illustrating chemical shift assignment. **a** Selected ^1^H-^13^Cα slices at the ^15^N chemical shifts of the residues Y154-E157 from the 3D HNCA spectra, demonstrating consistent sequential assignment of the residues in all states. **b** Superimposed expanded regions of 2D ^1^H-^15^N TROSY spectra showing chemical shift perturbations upon ligand binding. Residue labels for which correlations are shown in the slices from 3D HNCA spectra are highlighted in bold.

The TROSY-based 3D-HNCA experiments^32–34^ were recorded to perform the backbone ^1^H_N_ and ^15^N chemical shift assignment of the three MurD states (Fig. 1). In the BMRB database, only the ^1^H_N_ and ^15^N chemical shift assignment for the backbone of apo MurD was available, which was previously carried out by Saio et al.^27^ (BMRB entry 26641). It provided a good starting point for our assignment procedure, but could not be directly applied to MurD_apo_ spectra due to the different buffer composition required in our studies to fulfill comparable conditions for all three MurD states. Therefore, many ^15^N-^1^H_N_ correlation peaks in the spectra of MurD_apo_ were shifted in different directions with respect to the published assignments, and sequential assignment using 3D HNCA experiments was crucial to obtain the resonance assignment of all three MurD states. The chemical shift assignment was obtained for 75% of the MurD non-proline residues (Table S2). Mostly, the ^15^N-^1^H_N_ correlation signals of residues at the interface between CTD and CeD were absent in all states likely due to exchange broadening that could be attributed to the conformational exchange between open and closed MurD conformations, as previously shown by Saio et al.^27^.

**Fig. 2:**
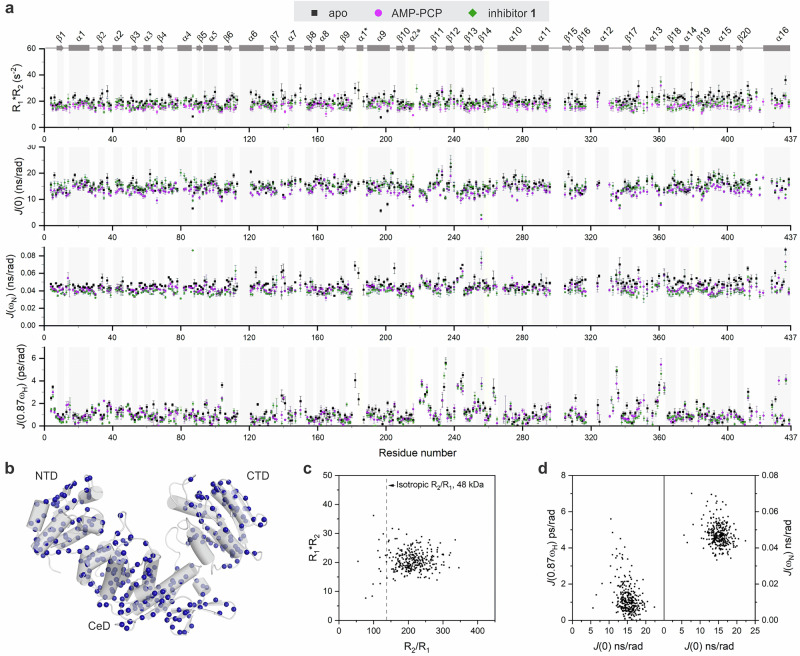
^15^N relaxation data and reduced spectral densities. **a** *R*_1_*R*_2_, *J*(0), *J*(*ω*_N_) and *J*(0.87*ω*_H_) for MurD_apo_ (in black) versus MurD_ACP_ (in magenta) and MurD_inh_ (in green). Secondary structure elements are presented and labeled as described by Bertrand et al.^21^. Helical turns that are observed in some X-ray structures, are marked with an asterisk. **b** ^15^N nuclei with available relaxation data (in blue spheres) for MurD_apo_ presented on X-ray structure of open conformation (PDB 1E0D^21^). **c** Plot of *R*_1_*R*_2_ versus *R*_2_/*R*_1_ for MurD_apo_. **d** Plots of *J*(*ω*_N_) and *J*(0.87*ω*_H_) as a function of the corresponding *J*(0) for MurD_apo_. The errors in the *R*_1_*R*_2_, *J*(0), *J*(*ω*_N_) and *J*(0.87*ω*_H_) values were estimated through error propagation.

The TROSY-based NMR experiments^35^ were conducted to measure the ^15^N spin-lattice relaxation rates (*R*_1_), ^15^N spin-spin relaxation rates (*R*_2_) and steady-state ^1^H-^15^N nuclear Overhauser enhancement (hetNOE) for MurD_apo_, MurD_ACP_, and MurD_inh_ (Fig. S1**)**. The ^15^N nuclei with available relaxation data were sufficiently distributed across each MurD state (Fig. 2b) to study the dynamics of the entire protein.

The distribution of ^15^N relaxation rates in plots of *R*_1_*R*_2_ versus *R*_2_/*R*_1_ (Fig. 2c) suggests that the three MurD states exhibit an anisotropic character^36^, therefore RSDM approach^37^ was utilized to characterize the frequency spectrum of the motions of N-H bond vectors. This approach allowed determination of reduced spectral densities at three different frequencies *J*(0), *J*(0.87*ω*_H_) and *J*(*ω*_N_) from relaxation rates and hetNOEs measured at a single spectrometer frequency, where *J*(0.87*ω*_H_) is approximated single spectral density term for linear combination of spectral densities at high frequencies (*ω*_H_, *ω*_H_ ± *ω*_N_)^37^ (Figs. 2a,  S2).

In general, increased *J*(0.87*ω*_H_), *J*(*ω*_N_) (Fig. 2a) and *R*_1_ values (Fig. S1) and lower hetNOE values (Fig. S1) were observed in the loop regions of MurD states, indicating local dynamics on the fast ps-ns (sub-nanosecond) time scale. The increased values of *R*_1_*R*_2_ and *J*(0) also found in α-helices and β-strands may indicate conformational exchange dynamics on the micro-to millisecond (μs-ms) timescale^36,38,39^. However, in order to draw conclusions about the time scale of specific dynamical events along the backbone of the three MurD states, the relationships between the three spectral densities need to be determined^38^.

The usual approach, applied to smaller proteins with few dynamically distinct regions^38^ extracts these relationships from the plots of *J*(*ω*_N_) and *J*(0.87*ω*_H_) as a function of the corresponding *J*(0). Moreover, for smaller proteins, it appears to be more straightforward to infer enhanced fast N-H bond motions from a high *J*(0.87*ω*_H_) and the presence of μs-ms exchange motions from a high *J*(0)^40^. However, for large proteins such as MurD, no specific correlation between the three densities corresponding to different dynamic properties can be recognized in these diagrams (Fig. 2d). Therefore, further details on the spectral densities, their correlations, and variations were sought for using PCA.

### Mechanistic insight into dynamics of MurD states through PCA of reduced spectral densities

In our analysis, the main purpose of PCA was not dimensionality reduction, but to determine how the spectral density terms vary together and how this reflects the co-operation of dynamics on different time scales. In interpreting the PCA results, we considered *J*(0.87*ω*_H_) and *J*(*ω*_N_) to be representative of the dynamics on the fast ps-ns time scale and *J*(0) to be representative of the dynamics on the ns-ms time scale, where μs-ms interval refers to chemical exchange as conformational exchange^41^. PCA yielded a non-negligible proportion of the variance explained by each of the three PCs (Table 1), with PC1 covering more than 55% in the three MurD states. The obtained PCs describe three independent dynamic mechanisms hidden in the source spectral density data. The moderate correlations between the spectral densities in Table 1 indicate the co-operation of the dynamics over different time scales, while the correlations between the spectral densities and the PCs show the extent to which the dynamics on the time scale representative of each *J*-term contribute to a particular dynamic mechanism.

**Table 1:**
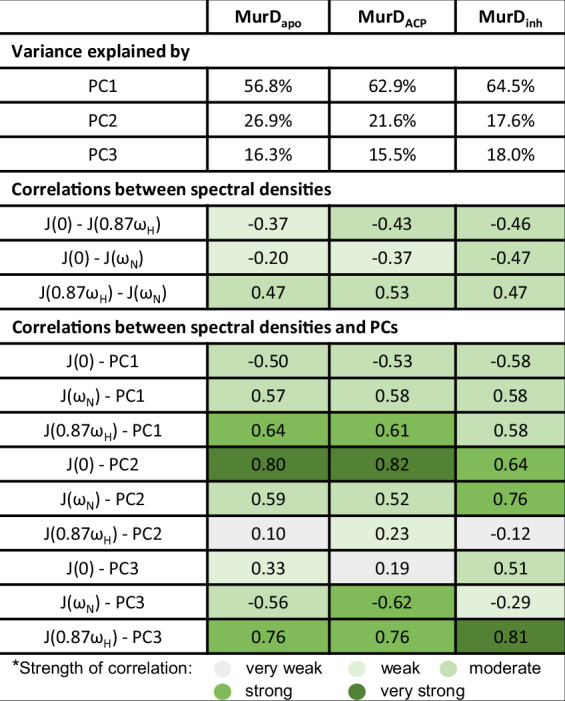
Summary of PCA

Proportion of variance explained by PCs, correlation between variables and correlation between variables and PCs for MurD_apo_, MurD_ACP_ and MurD_inh_.

The correlations between the three spectral densities in Table 1 appear to be more pronounced for MurD_ACP_ and MurD_inh_ than for MurD_apo_, but nevertheless remain only moderately correlated, so that each of the three PCs is an important source of information about the dynamics. The strengths and signs of the correlation between PC1 and the original variables suggest that the PC1 axis encodes the compensation effect between the dynamics on the fast ps-ns time scale and the time scale defined by *J*(0). The PC2 component contains similar information about the dynamics for the MurD_apo_ and MurD_ACP_ states, where the correlation of PC2 with *J*(0) predominates, while the situation is slightly different for the MurD_inh_ state, where the correlation of PC2 with *J*(*ω*_N_) becomes more important. Nevertheless, it can be summarized that the PC2 axis mainly encodes the cooperation of the dynamics on the sub-nanosecond time scale, characterized by *J*(*ω*_N_), and on the time scale defined by *J*(0). The PC3 component for the MurD_apo_ and MurD_ACP_ states shows the strengths and signs of the correlation to the original variables, indicating another compensation effect, in this case mainly between the dynamics on the sub-nanosecond time scales characterized by *J*(0.87*ω*_H_) and *J*(*ω*_N_). For the MurD_inh_ state, this compensation effect is no longer in the foreground as it diminishes and somehow turns towards the cooperative dynamics on the sub-nanosecond time scale characterized by *J*(0.87*ω*_H_) (i.e., the ps regime, see ^41^) and the time scale defined by *J*(0).

Overall, comparing the proportions of variance explained by the PCs for the three states shows that the compensation effect described by PC1 gains importance, while the dynamics described by PC2 contribute less to the variability in the residue dynamics upon binding of AMP-PCP or, more markedly, upon binding of inhibitor **1**. Moreover, the turnover of the percentage of variance explained by PC2 and PC3 for MurD_inh_ suggests that the dynamic mechanisms of this state are more distinct from the other two states.

**Fig. 3:**
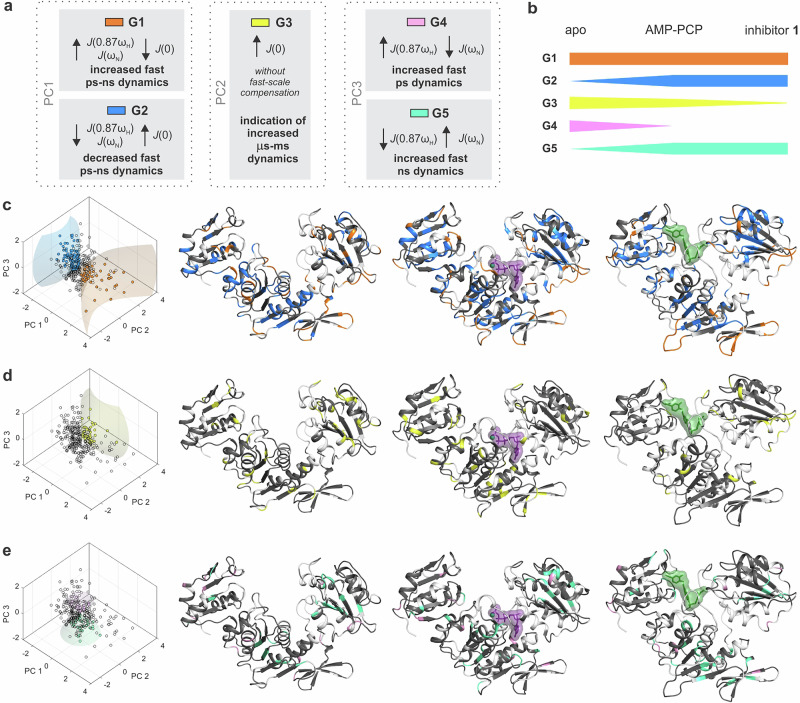
Categorization of residues into groups G1-G5 based on PC scores. **a** Legend defining groups G1-G5, each corresponding to a distinct dynamic mechanism, captured by the first three principal components derived from spectral density data. **b** Chart showing changes in numbers of residues assigned to each group when comparing the apo and ligand bound states. **c**–**e** Structural mapping of classified residues onto MurD crystal structures: G1 (orange), G2 (blue), G3 (yellow), G4 (pink), and G5 (green). Left panels show PC score plots derived from the standardized data on *J*(0), *J*(*ω*_N_) and *J*(0.87*ω*_H_) for MurD_apo_. Circles denote the data points for the PC scores, with colored circles indicating residues assigned to G1-G5 based on classification criteria (Table 2), while unfilled circles indicate residues not assigned to any group. Colored cones define the classification regions in PC space which comprise the residues of a particular group. Right panels display the crystal structures of MurD in three conformational states: the open conformation of MurD_apo_ (left, PDB 1E0D^21^), the semi-closed conformation with ATP (magenta) for MurD_ACP_ (middle, PDB 8VW2^22^), and the closed conformation with inhibitor **1** (forest green) for MurD_inh_ (right, PDB 2XPC^11^). Note that G4 and G5 group of residues for MurD_inh_ contains somewhat different information than for the other two states, as indicated above. Residues shown in grey have no group assigned, while residues shown in white indicate unavailable data.

Using the PC scores for each residue, we classified the residues into five groups G1-G5 based on the dominance of one PC over the other two PCs, i.e., considering one of the dynamic mechanisms to be dominant over the other two. The classification criteria can be found in the Materials and Methods section in Table 2. The dominance of PC1 over PC2 and PC3 refers to the dominance of the compensation effect between the dynamics on the fast ps-ns time scale and the time scale defined by *J*(0), which can occur in one or another way and determines groups G1 and G2. The dominance of PC2 over PC1 and PC3 shows the dominance of the non-compensation effect, which is only of interest with regard to the enhancement of this effect. Group G3 was determined in this way. The dominance of PC3 over PC1 and PC2 indicates the dominance of another compensation effect, which in turn is of interest in one or the other direction of compensation and determines the groups G4 and G5. The assignment of the residues to the groups on the basis of the selected criteria is exclusive, i.e., each residue can be assigned to at most one of the groups G1-G5 or none of them. The results of this classification are shown residue by residue in Table S3 and graphically in Fig. 3. The number of residues that are classified into a particular group for each MurD state is further summarized in Table S4, based on their listing in the groups in Table S3. For example, in Table S3, MurD_apo_ residues number 10, 56 and 89 are listed in group G5, resulting in a number of 3 in the corresponding MurD_apo_ NTD cell of Table S4.

**Table 2:** Criteria for the principal components (PC) scores determining the sets of residues of particular dynamics

Criteria	Description and interpretation	Corresponding group of data set in Fig. 3
${{{\rm{PC}}}}1 > \sqrt{{{{{\rm{PC}}}}2}^{2}+{{{{\rm{PC}}}}3}^{2}}$	Residues, where the decreased *J*(0) compensates for the increased *J*(0.87*ω*_H_) and *J*(*ω*_N_) (i.e., increased fast ps-ns dynamics) and this compensation effect prevails over the other two mechanisms (explained by PC2 and PC3)	G1, orange spheres in Fig. 3
$-{{{\rm{PC}}}}1 > \sqrt{{{{{\rm{PC}}}}2}^{2}+{{{{\rm{PC}}}}3}^{2}}$	Residues, where the increased *J*(0) compensates for the decreased *J*(0.87*ω*_H_) and *J*(*ω*_N_) (i.e., decreased fast ps-ns dynamics) and this compensation effect prevails over the other two mechanisms (explained by PC2 and PC3)	G2, blue spheres in Fig. 3
${{{\rm{PC}}}}2 > \sqrt{{{{{\rm{PC}}}}1}^{2}+{{{{\rm{PC}}}}3}^{2}}$	Residues, where *J*(0) (and less evidently *J*(*ω*_N_)) is increased without compensation on the time scale defined by *J*(0.87*ω*_H_) and this effect prevails over the other two mechanisms (explained by PC1 and PC3)	G3, yellow spheres in Fig. 3
${{{\rm{PC}}}}3 > \sqrt{{{{{\rm{PC}}}}1}^{2}+{{{{\rm{PC}}}}2}^{2}}$	Residues, where the decreased *J*(*ω*_N_) compensates for the increased *J*(0.87*ω*_H_) and this compensation effect prevails over the other two mechanisms (explained by PC1 and PC2)^#^	G4, pink spheres in Fig. 3
$-{{{\rm{PC}}}}3 > \sqrt{{{{{\rm{PC}}}}1}^{2}+{{{{\rm{PC}}}}2}^{2}}$	Residues, where the increased *J*(*ω*_N_) compensates for the decreased *J*(0.87*ω*_H_) and this compensation effect prevails over the other two mechanisms (explained by PC1 and PC2)^#^	G5, green spheres in Fig. 3

PC1 stands for the scores of the first principal component, PC2 for the scores of the second principal component and PC3 for the scores of the third principal component. The notation # in the table indicates a deviation from the given description for MurD_inh_, as explained in the text following Table 1.

We can consider the residues in the G1 group as those whose predominant dynamic mechanism is underpinned by the increased probability of dynamics on the fast ps-ns time scale due to the decreased probability of dynamics on the time scale determined by *J*(0). The G1 residues are typically found in loop areas and at the termini of α-helices and β-strands, mostly at the protein’s outer surface (Fig. 3c). Such behavior of *J*(*ω*) values is typical for the sub-nanosecond flexibility of the N-H bond vector^42^. The G2 group, on the other hand, combines residues with the predominant mechanism of sub-nanosecond flexibility reduction due to the increased probability of dynamics on the time scale determined by *J*(0). The G2 residues are mainly found in secondary structural elements and at the end(s) of the loops. The total number of G1 residues remains similar for the three MurD states, while the total number of G2 residues increases upon binding of either AMP-PCP or inhibitor **1** (see Fig. 3b and c,  S7, Tables S3 and S4 for details).

Group G3 represents the residues with the dominating mechanism underpinned by an increased probability of dynamics on the time scale determined by *J*(0) with a less intense but still present increase of probability of dynamics on the time scale determined by *J*(*ω*_N_), These are accompanied with the unchanged probability of dynamics on the ps time scale determined by *J*(0.87*ω*_H_). The total number of the residues with G3 dynamics most evidently changes upon binding of inhibitor **1** (see Fig. S7, and Table S4).

The other compensation effect within the sub-nanosecond time scale is captured by PC3 for MurD_apo_ and MurD_ACP_. This compensating mechanism diminishes for the MurD_inh_ state by turning towards the mechanism of cooperative dynamics as described above. The prevalence of PC3 over the other two PCs is given in the context of groups G4 and G5, whose meaning for the MurD_inh_ state is somewhat different from that of the other two states with regard to the mechanism described by PC3. The total number of residues classified in G4 is relatively low and spread without any particular pattern within the loops and secondary structures. The residues of group G5 turn out to be located in α-helices and β-strands as well as at the end(s) of the loops. The number of G4 residues decreases and the number of G5 residues increases upon binding of either AMP-PCP or inhibitor **1** (see Fig. S7, Tables S3 and S4 for details).

Further, we have analyzed the presence of residues of a particular group in the secondary structure elements, that we have defined as segments (such as a single α-helix, β-strand, or loop as provided in Table S3). The analysis shows that segments can contain residues from different groups. In the NTD domain for all three MurD states, about half of the segments consist of the residues belonging to only one of the five groups, together with the residues with non-significant dynamics (i.e., the residues that do not belong to any of the five groups). The other half of the segments contains the residues belonging to at least two of the five groups. In the CeD and CTD domains, there are about two-thirds of the latter and one-third of the former kind. Segments with residues belonging to different groups, i.e., the segments with PCA group variability, can be considered as with more complex dynamics, and the larger proportion of these segments supports the picture of greater complexity in the dynamics of the CeD and CTD domains.

Since the proportion of segments with greater dynamics complexity outweighs the segments with residues belonging to only one group, we assume that the segments with greater dynamics complexity bear more responsibility for the circumstances that influences the efficacy of the binding. These aspects are considered further in the following subsection together with the conformational exchange dynamics.

### Common aspects of conformational exchange dynamics and PCA results for MurD states

**Fig. 4:**
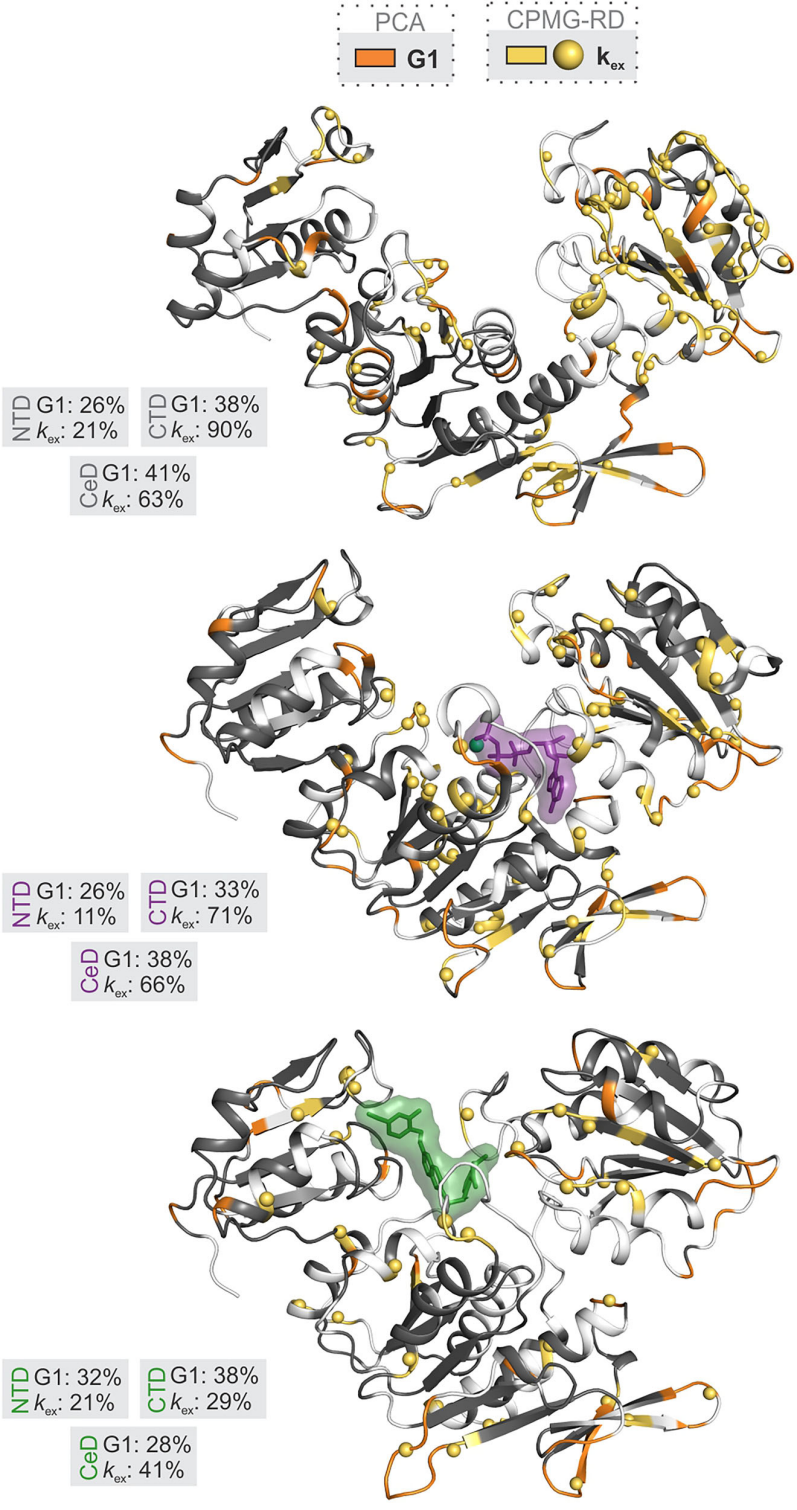
Distribution of the residues with conformational exchange and fast ps-ns dynamics. The residues with conformational exchange dynamics on the μs-ms time scale determined at 800 MHz with CPMG-RD are labeled as backbone in yellow and the amide nitrogen as yellow spheres, while the residues with increased fast ps-ns dynamics assigned to group G1 are labeled as backbone in orange. Yellow spheres on orange backbone indicate the amide nitrogen of residues with identified conformational exchange on μs-ms time scale and increased fast ps-ns dynamics. Gray boxes indicate the percentage of residues assigned to G1 and those with an exchange contribution (*k*_ex_) within each domain, as labeled. The same crystal structures^11,21,22^ are used to visualize these distributions for MurD_apo_ (upper), MurD_ACP_ (middle) and MurD_inh_ (lower), as explained in Fig. 3.

**Fig. 5:**
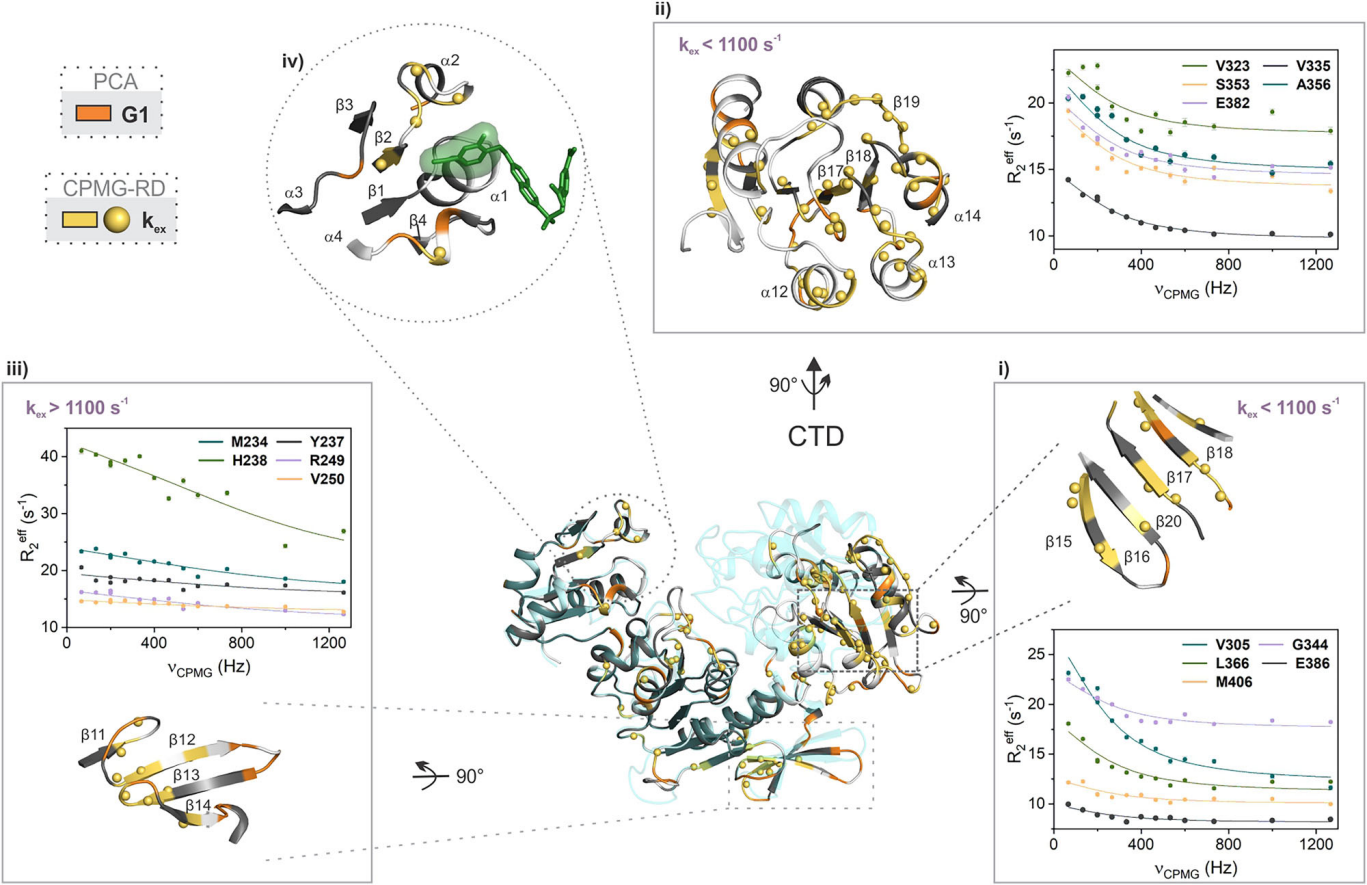
Excerpts of the MurD regions with a salient pattern of conformational exchange and fast ps-ns dynamics. The excerpted regions are shown for the MurD_apo_ on the X-ray structure of open conformation (PDB 1E0D^21^) with observed conformational exchange dynamics on μs-ms time scale identified by CPMG-RD (backbone in yellow and amide nitrogen in yellow spheres) and with increased fast ps-ns dynamics assigned to G1 group (backbone in orange). Yellow spheres on orange backbone indicate the amide nitrogen of residues with identified conformational exchange on μs-ms time scale and increased fast dynamics. Residues shown in white indicate unavailable data. The closed structure (in transparent cyan, PDB 2XPC^11^) is overlaid over CeD. Regions shown: i) β-sheet (β15-β16-β20-β17-β18-β19) in the CTD, (ii) CTD with highlighted helices (α12, α13 and α14) and loops (α12-β17, α13-β18 and α14-β19) (iii) β−meander (β12-β13-β14) in the CeD, (iv) uracil-binding pocket (β1, β2, β1-α1, β2-α2, β3-α3 and β4-α4) with a potentially bound inhibitor shown in green. Representative CPMG-RD plots are shown, illustrating μs-ms dynamics (*k*_ex_) for residues from each region. *R*_2_^eff^ represents the effective transverse relaxation rate at each refocusing pulse train frequency (*ν*_CPMG_). The errors in the *R*_2_^eff^ values were determined based on the uncertainties of the peak intensities estimated from the background noise level of the spectra.

The conformational exchange dynamics on the μs-ms time scale of the three MurD states were further investigated using TROSY-based ^15^N Carr–Purcell–Meiboom–Gill relaxation dispersion (CPMG-RD) NMR method^43–45^. The presence of conformational exchange dynamics was assessed based on the relationships between the experimental data and the data fitted with the 2-site CPMG model as derived and provided by Korzhnev et al.^46^. The residues with reliable evaluation of the presence of conformational exchange, as explained in the Materials and Methods section, are listed in Table S3 and with the exchange rates from the fitting in Table S5 and shown in Figs. 4 and 5.

Screening the possible overlaps between the detected conformational exchange dynamics on the μs-ms time scale and the dynamic events recognized by PCs and the groups, we first encountered the following. We found that in MurD_apo_, about 60% of the segments with conformational exchange are the segments containing residues belonging to at least two different groups. This ratio increases to over 70% in the MurD_ACP_ and MurD_inh_ states, which is due to the increase of these segments in their CeD and CTD domains. Therefore, evidence of conformational exchange for the residues in a given segment predicts with a probability of at least 60–70% the diversity of dynamics expressed by the presence of different groups in that particular segment.

On the other hand, we wanted to investigate the possibility of predicting conformational exchange dynamics on the μs-ms time scale based on information from PCA group classification. We took into account that conformational exchange should be reflected in an increased *J*(0) without compensation effect. PC2 appeared to be the most promising indicator with a strong positive correlation with *J*(0). However, it is also positively correlated with *J*(*ω*_N_), so that a limited predictive ability was to be expected. The segment was classified as having possible conformational exchange dynamics if it consists of at least one residue for which PC2 predominates over the other two PCs, i.e., of at least one residue belonging to G3 group. As shown in Table S6, the G3 group defined by PCA shows the best overall agreement with the observation of conformational exchange dynamics using the CPMG-RD method. Predictions using elevated values of *R*_1_*R*_2_ products are slightly less reliable, while the approximate approach to determine *R*_ex_^36^ is the least reliable for predicting the conformational exchange dynamics of MurD states. The best agreement between G3 and CPMG-RD per segment resolution was obtained for MurD_apo_, which became evidently reduced for MurD_inh_. This could be due to a lower number of available data for MurD_inh_ together with a slightly changed role of PC2, where its correlation with *J*(*ω*_N_) starts to dominate its correlation with *J*(0). Nevertheless, the prediction of both the presence and absence of conformational exchange dynamics based on the G3 residues gave 65–75% agreement with the CPMG-RD results per segment resolution.

### Flexibility of MurD states from the perspective of conformational exchange and fast time scale dynamics

The results of Kumar et al.^47^ showed how fast ps-ns motions and μs-ms conformational exchange dynamics of protein kinase p38 control its regulation and activation. Inspired by this, we similarly investigated the distribution pattern of residues with conformational exchange dynamics on the μs-ms time scale and of residues with the identified increased fast ps-ns dynamics (G1 residues). Indeed, this revealed an additional perspective on the ability (flexibility) of MurD to adapt its structure upon binding of a ligand.

The number of segments with conformational exchange is significantly lower in NTD than in the other two domains for all three states. While conformational exchange is represented to a similar extent in the CeD and CTD of MurD_apo_ and MurD_ACP_, it gets reduced significantly in MurD_inh_, especially in the CTD (Tables S3 and S4, Fig. 4). These observations suggest that the main process underlying the conformational exchange can be attributed to the movements of the CTD relative to CeD, which are not suppressed upon binding of AMP-PCP, but upon binding of inhibitor **1**. The extent of the segments with G1 dynamics (Table S3, Fig. 4) is more evenly distributed across the domains for all three states. Their number overweight the number of segments with conformational exchange only in the NTD of all states and in the CTD of MurD_inh_.

The *k*_ex_ values obtained by the fit per residue for MurD_apo_ (Table S5, Fig. 5) were considered as clues to identify subsets of structurally related residues that have a similar range of *k*_ex_. For the identified subsets of residues, we performed a group fit and characterized two different global conformational exchange processes on the μs-ms time scale. One subset of residues is located in the CTD with *k*_ex_ values below 1100 s^−1^, suggesting a conformational exchange that may be related to the CTD motion. For example, these *k*_ex_ values are observed for contact residues within the β-sheet (β15-β16-β20-β17-β18-β19) in the outer region of CTD (Fig. 5, i), which approach the CeD in the open structure, and residues at the termini of helices α12, α13 and α14, as well as, for several residues in the α12-β17 loop, which approach the CeD in the closed structure (Fig. 5, ii). Residues with *k*_ex_ below 1100 s^−1^ from these regions were group fit resulting in a group *k*_ex_ of 836 s^−1^ and *p*_A_ of 0.5. At the same time, we can observe the presence of fast ps-ns dynamics in the outer loops of CTD adjacent to the above structures with the synchronized conformational exchange, i.e., in the loops α12-β17, α13-β18 and α14-β19.

The other subset of residues is located in the CeD and exhibits various *k*_ex_ values above 1100 s^−1^. It characterizes the motion of a β-meander (β12-β13-β14) and adjacent loops β11-β12 and β13-β14 (Fig. 5, iii) positioned away from ligand binding sites, not facing the CTD. The group fit for the residues with *k*_ex_ above 1100 s^−1^ from the β-meander and adjacent loops resulted in a group *k*_ex_ of 2514 s^−1^ and *p*_A_ of 0.97. Interestingly, the residues near the β-meander, specifically in the β12-β13 and β14-α10 loops, which approach the CTD in the open structure, exhibit fast dynamics on the ps-ns time scale (Fig. 5, iii).

In the MurD_ACP_, the residues with *k*_ex_ values below 1100 s^−1^ dominate in the CTD in the above-mentioned β-sheet (Table S5, Fig. 4), and yield a group *k*_ex_ of 486 s^−1^ and *p*_A_ of 0.53. In addition, the total number of segments in the CTD domain with the residues in conformational exchange does not reduce significantly. This suggests that the motion of the CTD on the μs-ms time scale is mainly preserved. We can speculate that the binding of AMP-PCP does not lock the conformation to any of those observed in the X-ray structures of MurD bound to substrates or inhibitors. This would imply that an ensemble of conformations is still available for subsequent substrate binding. The main reduction in the number of residues with conformational exchange within the β-sheet and overall in the CTD of MurD_inh_ compared to the other two states indicates a stabilization of this domain upon binding of inhibitor **1**. The fast ps-ns dynamics remains present in the loops α12-β17 and α13-β18 in both MurD_ACP_ and MurD_inh_.

In the β-meander (β12-β13-β14) of MurD_ACP_ and MurD_inh_, the number of residues with *k*_ex_ decreases. In the MurD_ACP_ state, the values remain mainly in the range above 1100 s^−1^ from the per-residue fit, while the group fit for these residues gives a group *k*_ex_ of 2364 s^−1^ and *p*_A_ of 0.5. The *k*_ex_ values in the β-meander of MurD_inh_ cover the ranges below and above 1100 s^−1^ (Table S5, Fig. 4). The fast ps-ns dynamics is preserved in the loops β12-β13 and β14-α10 upon binding of AMP-PCP and inhibitor **1**.

While the conformational exchange dynamics changes significantly in the CeD and CTD of MurD_inh_, the fast ps-ns dynamics is preserved in the β12-β13, β13-β14 and β14-α10 loops of CeD and the α12-β17 and α13-β18 loops of CTD in all three states (Table S3, Fig. 4). These are the loops surrounding the substructures with a particular conformational exchange dynamic, as described above. Thus, it appears the loops facilitate the fast time scale flexibility of these substructures (their adaptability to the environment), which is not switched off when the ligand is bound.

In the NTD of all three states, the residues with identified conformational exchange are mainly located in the segments that form the uracil binding pocket (Fig. 4 and Fig. 5, iv), which is part of the inhibitor **1** and UMA binding sites. Their *k*_ex_ values extend over several scales and cannot be attributed to any overall motion of NTD. In addition, there is no particular pattern of fast ps-ns dynamics in the NTD domain. This speaks in favor of impoverished flexibility of the NTD domain in all three states.

**Fig. 6:**
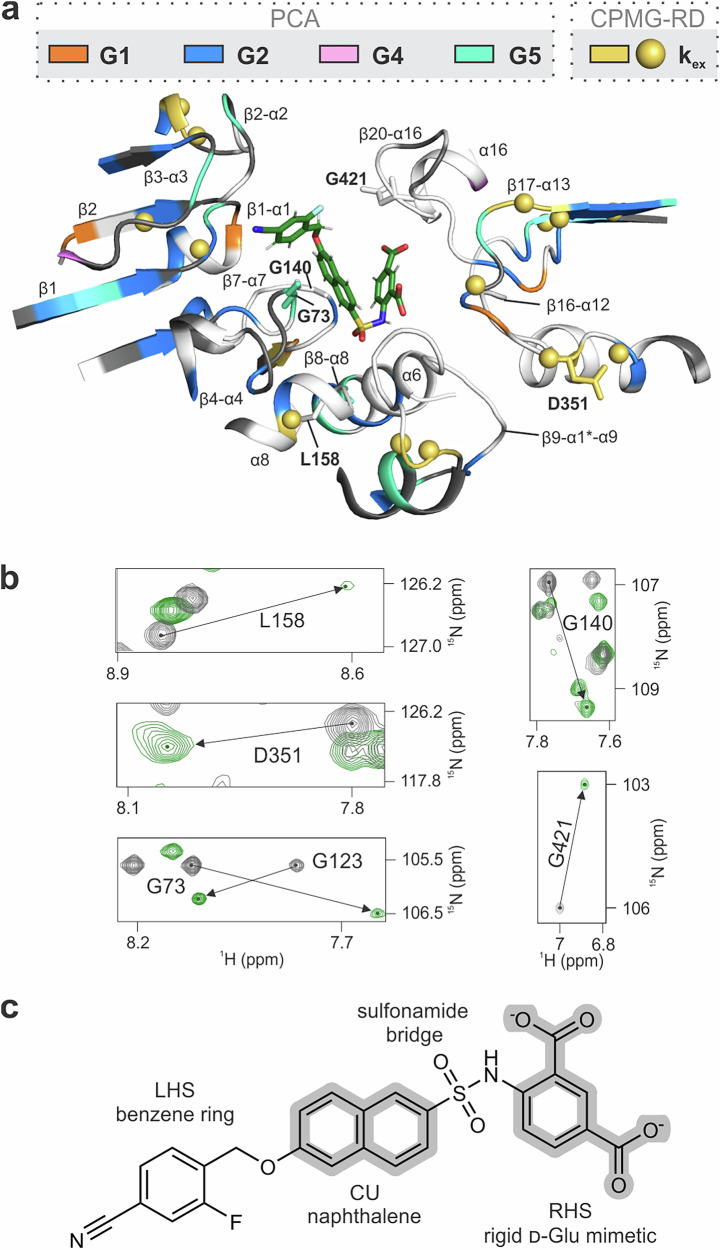
Dynamics of the inhibitor 1 binding site. **a** The inhibitor **1** binding site as determined by Simčič et al.^20^ highlighting residues with distinct dynamic mechanism according to PCA (G1 in orange, G2 in blue, G4 in pink, and G5 in cyan) and/or conformational exchange dynamics on μs-ms timescale as identified by CPMG-RD (yellow backbone and sphere). Residues shown in grey indicate residues with no group assigned, while residues in white indicate unavailable data. **b** Overlaid ^1^H-^15^N TROSY spectra of MurD_apo_ (gray) and MurD_inh_ (green) are shown for residues in the ligand-binding site that exhibit the strongest perturbations upon the addition of inhibitor **1** added in 4 equivalents. (**c**) Chemical structure of inhibitor **1** (4-(6-(4-cyano-2-fluorobenzyloxy)naphtalene-2-sulfonamido)isophthalic acid) with labeled structural moieties. LHS: Left-hand side, CU: Central unit, RHS: Right-hand side.

Inhibitor **1** interacts with all three domains via 15 segments (Table S3, Fig. 6). The dynamic behavior of these segments is not uniform in any of the three states. In MurD_apo_, many of the segments exhibit vivid dynamics that is largely conserved in MurD_ACP_ (Table S3). Although the proportion of binding segments in conformational exchange and the variability of the PCA group are significantly reduced in MurD_inh_ (Table S3), none of the four inhibitor **1** moieties (Fig. 6c) is exclusively surrounded by stable or flexible segments (Fig. 6a). We can still observe conformational exchange along with PCA group variability (presence of residues belonging to at least two of the PCA groups) at the inhibitor **1** binding sites of the β1-α1 and β2 segments in the NTD, β17-α13 and β20-α16 in the CTD and conformational exchange of α8 and β9-α1*-α9 in CeD (Fig. 6a, Table S3). We believe that these findings support the limited binding efficacy of inhibitor **1** and its analogs^10,11,26^ and call for fine-tuning of each moiety of these types of inhibitors to achieve better adaptability of these binding segments.

### Common aspects of MD simulations and experimental findings for MurD states

The agreement between the results of all-atom MD simulations performed for three systems (MurD_apo_, MurD_ATP_, and MurD_inh_), each of the simulation length 2 μs, and the experimental data per segment was sought for using the criteria in Table 3. For example, if the MD results identified at least one residue in a particular segment that met the first criterion in Table 3, and at the same time the analysis of the experimental results ranked at least one residue in group G1 for that particular segment, we considered this as a match between MD prediction and experimental results. In the same way, we looked for agreement between the MD results fulfilling the second criterion in Table 3 and the presence of G2 residue(s) in a given segment, and so on. In this regard, the prediction of the MD simulation results for the presence of the G1 group at the segment resolution was relatively high (61 – 84%), as well as for the presence of the G2 group (76 – 91%) and CPMG-RD (62 – 82%). The agreement of the simulations with the presence of the G4 and G5 groups was mostly below 50%.

**Table 3:** Residue classification criteria based on the MD spectral density data

Criteria for the classification of the residues (*Res*) based on the MD spectral density data	Results based on the experimental data to be compared to the MD results
$({d}_{1,{Res}} < 0){{{{\rm{and}}}}}({d}_{2,{Res}} > 0)$	G1
$\left({d}_{1,{Res}} > 0\right){{{{\rm{and}}}}}({d}_{2,{Res}} < 0)$	G2
$({d}_{3,{Res}} < 0){{{{\rm{and}}}}}({d}_{4,{Res}} > 0)$	G4
$\left({d}_{3,{Res}} > 0\right){{{{\rm{and}}}}}({d}_{4,{Res}} < 0)$	G5
${d}_{1,{Res}} > 0$	CPMG-RD

Comparison with the experimentally determined G1, G2, G4 and G5 and CPMG-RD groups. The differences *d*_*1,Res*_, *d*_*2,Res*_, *d*_*3,Res*_ and *d*_*4,Res*_ result from Eq. (13)

**Fig. 7:**
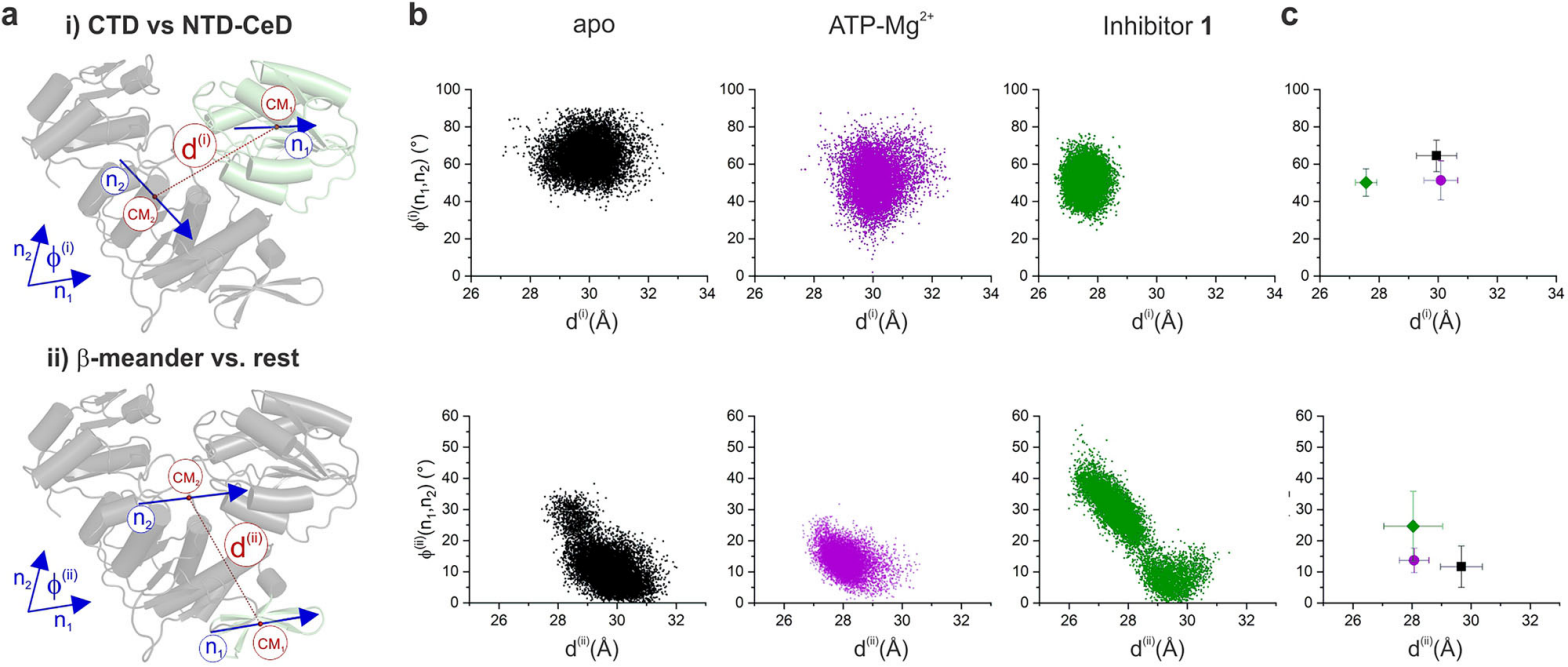
Relative movements between the selected subdomains determined by the MD simulation as fluctuations in the distance between the centers of the vectors and the angle they enclose. **a** Schematic representation of the selected pairs **i**) and **ii**) of subdomains with the corresponding direction vectors (**n**_**1**_ and **n**_**2**_), centers of vectors (as centers of mass, CM_1_ and CM_2_), distances between CMs (*d*^(i)^ and *d*^(ii)^) and angles (*ϕ*^(i)^ and *ϕ*^(ii)^) determined. **b** Angle distribution versus center distance and (**c**) centers of the data point clouds with standard deviations in the distance and angle of MurD_apo_(black), MurD_ATP_(magenta), and MurD_inh_ (green).

Thus, the established criteria for MD-based residue classification capture well the main dynamic mechanism described by PC1 of experimentally determined spectral densities, as the percentage of agreement with G1 and G2 is relatively high. In addition, MD successfully predicts conformational exchange per segment resolution to a similar extent. Continuing from this observation we further looked into MD simulations of the relative movements between the selected subdomains of MurD. Namely, between CTD versus NTD-CeD and between β-meander versus the rest as some specific peculiarities for these movements were identified from the experimental data as given in subsection 2.4. Figure 7 displays distributions of two-dimensional vectors representing structural arrangement during 2 μs MD simulations with a first component as the distance between the regions and a second as the relative orientation between the regions in the form of cloud patterns.

The distances *d*^(i)^ and *d*^(ii)^ between the regions (Fig. 7a, i and ii) are shown in Figure S8 as a function of simulation time, indicating more noticeable modifications in the dynamics upon binding of inhibitor **1**. Further details can be seen from the data clouds in Fig. 7b. The cloud patterns capture dynamic events on a μs time scale and it is therefore to be expected that faster motions are only brought forward when slower motions decrease. Against this background, a similarly dispersed relative movement of the CTD domain relative to NTD-CeD in the MurD_ATP_ state compared to MurD_apo_ (Fig. 7b, i) supports the essentially conserved conformational exchange dynamics observed in the experiment in the CTD domain of MurD_ACP_. On the other hand, the dispersion cloud for the MurD_inh_ state is shifted and reduced in size (Fig. 7b, i). This fits with the experimental findings of a significantly reduced number of residues with conformational exchange in the CTD domain and brought out increased G2 dynamics upon binding of inhibitor **1**, both indicating stabilization of this domain.

Figure 7b, ii shows a reduced dispersion of the β-meander dynamics versus the rest for MurD_ATP_ and an increased dispersion of the β-meander dynamics versus the rest for MurD_inh_ compared to MurD_apo_. This observation corroborates experimental data showing that the number of residues with conformational exchange in the β-meander decreases for MurD_ACP_, with exchange rates similar to those of MurD_apo_ (in the range above 1100 s^−1^) and no obvious changes in G1 and G2 dynamics. In contrast, the scattered green cloud in Fig. 7b, ii from the simulations supports the picture of the dynamics diversity of the β-meander in MurD_inh_. Although the conformational exchange is also reduced in the β-meander of MurD_inh_, the exchange rates become versatile and cover different ranges (below and above 1100 s^−1^). The reduction in conformational exchange brings out more evidently concentrated G1 dynamics in the vicinity of the β-meander together with basically preserved G2 dynamics compared to MurD_apo_.

## Discussion

Typically, relationships between reduced spectral density terms are visualized as *J*(0) vs *J*(*ω*_H_) and *J*(*ω*_N_), where, *J*(0) versus *J*(*ω*_H_) are negatively correlated while *J*(0) versus *J*(*ω*_N_) are positively or negatively correlated in dependence of the size of the protein. However, these plots do not simultaneously account for the interdependence of all three *J*-terms. In our analysis using PCA, we were able to recognize the interplay between them. The main strength of the PCA performed on the spectral density data is the revelation of dynamic mechanisms underlying the protein dynamics with per residue resolution, which aids the interpretation of the interplay between protein size, dynamics, and local structural context. Each of the three PCs corresponds to a specific mechanism defined by its dependence on particular spectral density terms. The classification of residues into five groups based on PCs was crucial for analyzing the dynamics of MurD comprising 430 non-proline residues. It allowed the grouping of residues with distinct dynamics, expressed as the interplay of events on different time scales, without relying on subjective classifications or biased thresholds, on which there is often no uniform consensus in the scientific community. On this basis, we were able to investigate the mechanistic differences between distinct conformations of the MurD states. Indeed, the mechanism of increased dynamics on the fast ps-ns time scale compensated by decreased dynamics on the time scale determined by *J*(0) described by group G1 does not differ significantly among the three MurD states. In contrast, the opposite compensation described by G2 shows that this mechanism extends over more segments in the MurD_ACP_ and MurD_inh_ states compared to MurD_apo_. The other compensation mechanism observed between the sub-nanosecond time scales and described by groups G4 and G5 is less pronounced, but still indicates an altered prevalence of these dynamics upon binding of either AMP-PCP or inhibitor **1**. In addition, we observed that the total number of residues with G3 dynamics describing the non-compensation mechanism of increased dynamics on the time scale determined by *J*(0) obviously changes upon inhibitor **1** binding.

Investigation of the ability of the PCA results to predict conformational exchange on μs-ms time scale proved limited at residue resolution, but relatively successful at segment resolution, as the prediction of both the presence and absence of conformational exchange based on the G3 residues showed 65–75% agreement with the CPMG-RD results. This is obviously better than the prediction based on the *R*_1_*R*_2_ products, especially in predicting the absence of conformational exchange (Table S6). Thus, the PCA results provide valuable information about these important motions that are directly linked to biological function^48^, even for larger proteins with complex dynamic behavior on multiple time scales, such as MurD.

The independent measurements of conformational exchange dynamics with CPMG-RD indicate the existence of a conformational ensemble through slow μs-ms movements of CTD in MurD_apo_ and MurD_ACP_, which are only suppressed after binding of inhibitor **1**. This suggests that AMP-PCP selects compatible conformations pre-existing in the apo ensemble and maintains the flexibility of the CTD to prepare the conformational ensemble favorable for substrate binding through a conformational selection process. Interestingly, 2 μs long MD simulations for MurD_apo_ and MurD_ACP_ also show that the relative flexibility of the CTD versus the rest is not that much different for the two states, while it is significantly lower in the case of MurD_inh_. Nevertheless, recent crystallographic structures of apo and bound MurD showed that the closed conformation can also exist in the AMP-PNP-bound state (PDB 9DQW^49^) and the semi-closed conformation in the apo state (PDB 5A5E^23^), suggesting that occupancy of the different conformations in the ensemble may be present at a higher percentage, that is in line with our predictions from the analysis of CPMG-RD data.

The prevalence of conformational selection in the binding mechanism may be the reason for the unsuccessful design of ATP-competitive inhibitors, which was based on a specific/single crystallographic structure in the closed conformation. To effectively target the ATP-binding site, we recommend generating an ensemble of conformations that includes only those in which the site remains exposed, excluding closed conformations.

A high degree of dynamic diversity observed through group classification that inhibitor **1** cannot fully suppress could be a key factor limiting its binding efficacy. However, we should bear in mind that increasing the target flexibility in the bound state by incorporating appropriate functional groups into the ligand may have the beneficial effect of improving the lifetime of a protein-drug complex and consequently increasing the drug efficacy^50^. In any case, the group classification based on PCs reveals details of less and more flexible protein dynamics and can therefore serve as an advanced guide for inhibitor design.

Of note, the approach of applying PCA to the reduced spectral densities is by no means limited to the MurD system, but represents a general tool that enables a deeper insight into dynamic mechanisms specific to any other system for which spectral density data are available.

## Materials and methods

### Protein expression and purification

Cultures of BL21(DE3) *Escherichia coli* containing the pABD16 plasmid for MurD expression were cultivated on LB agar plates for 16 h at 37 °C. For preparation of U-[^2^H, ^15^N]-labeled MurD, a single colony was transferred to 20 ml of LB medium prepared with 70% ^2^H_2_O and incubated overnight at 37 °C. Cells were subsequently pelleted, resuspended, and added to U-[^2^H, ^15^N]-labeled rich OD2 medium from Silantes. For U-[^2^H, ^13^C, ^15^N]-labeled MurD, single colony was transferred to 20 ml of LB medium and incubated overnight at 37 °C. After pelleting, the cells were resuspended in a series of adaptation media with gradually increasing ^2^H_2_O ratios: M9 prepared in H_2_O, M9 with 50% ^2^H_2_O and M9 with 100% ^2^H_2_O, each at a small scale. The adaptation process started with OD_600_ = 0.25 and continued until reaching ~2. Finally, the cells were resuspended in large-scale M9 medium with 100% ^2^H_2_O. Each M9 minimal medium contained U-[^2^H, ^13^C]-glucose (2 g l^−1^) and ^15^NH_4_Cl (1 g l^−1^). The culture was grown at 37 °C until an OD_600_ of 0.7 was reached. The temperature was then decreased to 25 °C, and after a 30-min, IPTG was added to 1 mM final concentration. After 16 h of induction, cells were harvested by centrifugation and resuspended in lysis buffer (50 mM Tris-HCl, pH 8 at 4 °C, 500 mM NaCl, 5 mM MgCl_2_, 20 mM imidazole, protease inhibitors (Sigma Aldrich), and 2 mM TCEP). The lysate was treated with lysozyme, sonicated, and after a 15 min incubation with benzonase, clarified by centrifugation at 12,000 rpm for 30 min at 4 °C. The supernatant containing the His-tagged protein was loaded onto a His-Trap column (GE Healthcare) equilibrated with binding buffer (50 mM Tris-HCl, pH 8 at 4 °C, 500 mM NaCl, 5 mM MgCl_2_, 20 mM imidazole, and 2 mM TCEP). Following extensive wash with the binding buffer, the protein was eluted using elution buffer (50 mM Tris-HCl, pH 8 at 4°C, 500 mM NaCl, 5 mM MgCl_2_, 300 mM imidazole, and 2 mM TCEP). To exchange ^2^H for ^1^H at amide groups in regions hidden from the solvent, the protein was first unfolded by dialysis against an unfolding buffer (50 mM Tris-HCl, pH 8 at 4 °C, 6 M GuHCl, 150 mM NaCl, 5 mM MgCl_2_, and 3 mM DTT) and then refolded by dialysis against a refolding buffer (50 mM Tris-HCl, pH 8 at 4 °C, 150 mM NaCl, 5 mM MgCl_2_, and 3 mM DTT). For the second purification step involving size-exclusion chromatography, the protein was dialyzed against GF buffer (20 mM Tris-HCl, pH 7.5 at room temperature, 130 mM NaCl, 5 mM MgCl_2_, and 3 mM DTT) and then loaded onto a 120 mL Superdex 75 PG column (GE Healthcare) pre-equilibrated with GF buffer. The purity and molecular weight of the eluted protein were assessed using SDS-PAGE. Pure fractions were combined, concentrated, and dialyzed against NMR buffer (20 mM Tris-*d*_6_-HCl, pH 7.2 at room temperature, 100 mM NaCl, 5 mM MgCl_2_, 1 mM TCEP, and 0.02% NaN_3_). The U-[^2^H, ^15^N]-labeled MurD was flash-frozen in liquid nitrogen and stored at −80 °C.

Compound **1** was resynthesized according to the synthetic procedures described in Sosič et al.^11^. The ^1^H and ^13^C NMR spectra were in accordance with previously published data. Compound had > 97% purity by HPLC.

### NMR spectroscopy

All experiments were recorded at 25 °C on a Bruker Avance Neo spectrometer operating at 18.8 T (800 MHz for ^1^H), and equipped with a ^1^H/^13^C/^15^N cryoprobe. Data acquisition was carried out using the pulse sequences from the Bruker’s library of pulse programs. The NMR samples were placed in 5 mm NMR Shigemi tubes and contained 0.27 mM U-[^2^H, ^15^N] or U-[^2^H, ^13^C, ^15^N]-labeled MurD in an NMR buffer, supplemented with 10% DMSO-*d*_6_.

#### Chemical shift assignment

The TROSY-based 3D-HNCA spectra were acquired with 32 scans using non-uniform sampling along the two indirect dimensions t_1_ and t_2_ containing 170 and 76 complex data points, respectively, of which only 6460-point pairs were recorded. This corresponded to an undersampling of 50%. Spectral widths 27 ppm (^13^C), 35 ppm (^15^N) and 14.2 ppm (^1^H), acquisition times 7 ms (^13^C), 30 ms (^15^N) and 45 ms (^1^H) and recycle delay of 1 s were used. The spectra were processed with Topspin 4.1.7. and analyzed with CCPNmr Assign 3.1.0^51^. The backbone ^1^H_N_ and ^15^N chemical shift assignments of MurD_apo_, MurD_ACP_, and MurD_inh_ are shown in the ^1^H-^15^N TROSY spectra (Figs. S3-S5).

#### Chemical shift perturbations (CSPs)

CSPs were tracked in ^1^H-^15^N TROSY spectra recorded with acquisition times of 45 ms (^15^N) and 90 ms (^1^H), 16 scans and a relaxation delay of 1 s. CSPs were calculated from the proton and nitrogen CSP (Δ*δ*_H_ and Δ*δ*_N_, respectively), according to the weighted formula for the *i*^th^ residue^52^:

1 $${{{{\rm{CSP}}}}}_{i}=\sqrt{{\left(\Delta {\delta }_{{Hi}}\right)}^{2}+0.14{\left(\Delta {\delta }_{{Ni}}\right)}^{2}}$$

CSP’s at binding of AMP-PCP (Fig. S6) were quantified at a protein-ligand ratio of 1:8. Subsequently, inhibitor **1** was titrated from a stock prepared in DMSO-*d*_6_. Final protein-inhibitor **1** ratio used for calculation of CSPs was 1:4 (Fig. S6). The addition of the inhibitor **1** did not exceed 2% of the NMR sample volume.

In most cases where a significant CSP was observed, the intensity of the unbound protein signal decreased with increasing ligand concentration and the new protein signal appeared with increasing intensity. The CSP could not be determined if the intensities of the new peaks were too low to obtain correlations in the HNCA spectra that would allow unambiguous assignment of the new peaks. The CSP data in the presence of AMP-PCP and inhibitor **1** indicate that MurD preferentially forms a complex with inhibitor **1** and not with both ligands. Namely, an additional set of new signals with lower intensity was observed in the ATP-binding region, also indicating the presence of a complex with both ligands. MD simulations confirmed these observations, as the complex of MurD with inhibitor **1** along the 2 μs long trajectory is far more stable and well-defined than the complex with both ligands.

#### ^15^N relaxation

The ^15^N *R*_1_ and *R*_2_ and hetNOE measurements^35^ for MurD_apo_, MurD_ACP_, and MurD_inh_ were acquired with acquisition times of 45 ms (^15^N) and 90 ms (^1^H). The *R*_1_ values were determined from spectra recorded with eight relaxation delays in the following sequence: 20, 3000, 60, 2000, 160, 1280, 320, 640, and 20 ms. The recycle delay was 6 s. The *R*_2_ values were determined from spectra measured with six Carr-Purcell-Maiboom-Gill (CPMG) loop lengths in the following sequence: 10, 50, 16, 39.9, 25, 29.9 and 16 ms. The recycle delay was 1 s. 16 scans were performed for *R*_1_ and 32 scans for *R*_2_ measurements. The estimation of the experimental errors in the *R*_1_ and *R*_2_ values was determined based on the deviation between duplicated spectra. The hetNOE values were determined from two datasets, one acquired with and the other without initial proton pre-saturation. A long pre-saturation/relaxation time of 10 s was used to consider long amide ^1^H *T*_1_ values in perdeuterated proteins^53^. 16 scans were performed for MurD_apo_ and MurD_ACP_ and 32 scans for MurD_inh_. The errors in the hetNOE values were determined based on the uncertainties of the peak intensities estimated from the noise level of the spectra, as described by Farrow et al.^54^. The *R*_1_ and *R*_2_ spectra were zero-filled to 8k in ^1^H dimension and processed using Topspin 4.1.7. Relaxation series were subsequently analyzed using CCPNmr Analysis 2.5.0^55^. Relaxation rates *R*_1_ and *R*_2_ were determined using non-linear least-squares fitting, where the peak intensities were fit to the equation:

2 $$I={I}_{0}{e}^{\left(-{Rt}\right)}$$

In this equation, both *I*_0_ and *R* were treated as fit parameters.

The reduced spectral densities *J*(0), *J*(0.87*ω*_H_) and *J*(*ω*_N_) were calculated from *R*_1_, *R*_2_, and hetNOEs using the program Relax (Version 5.0.0)^56,57^. The vibrationally averaged effective N-H bond length was 1.02 Å and ^15^N chemical shift anisotropy was −172 ppm.

The ^15^N CPMG-RD experiments^43–45^ were acquired using a constant CPMG relaxation delay (*T*_relax_) of 30 ms. The CPMG filed strengths (*ν*_CPMG_) were: 67, 133, 200 (2×), 267, 333, 400, 467, 533, 600, 733, 1000, and 1267 Hz with duplicate points included for error analysis. The spectra were measured with 24 scans and a recycle delay of 3 s. The spectra were zero-filled to 8k in ^1^H dimension and processed using Topspin 4.3.0 and CCPNmr Assign 3.1.0^51^. Effective transverse relaxation rates (${R}_{2}^{{{{\rm{eff}}}}}$) were calculated from the peak intensities at given CPMG frequency (*I*_*ν*_) relative to the intensity at *ν*_CPMG_ = 0 Hz (*I*_0_) using the equation:

3 $${R}_{2}^{{{{\rm{eff}}}}}=-\frac{1}{{T}_{{{{\rm{relax}}}}}}{{{\mathrm{ln}}}}\frac{{I}_{\nu }}{{I}_{0}}$$

${R}_{2}^{{{{\rm{eff}}}}}$ were then plotted as a function of *ν*_CPMG._

The precision of the peak intensities at a given *ν*_CPMG_ (i.e., *I*_*ν*CPMG_) and intensity at *ν*_CPMG_ = 0 Hz (i.e., *I*_0_) was determined as the mean of the background noise obtained from the random sampling for each *ν*_CPMG_, i.e., Δ*I*_*ν*CPMG_ and Δ*I*_0_ (for *ν*_CPMG_ = 0 Hz).The error bars for the data points of ${R}_{2}^{{{{\rm{eff}}}}}$ in s^−1^ were further calculated as

4 $$\triangle {R}_{2}^{{{{\rm{eff}}}}}\left({\nu }_{{{{\rm{CPMG}}}}}\right)=\pm \frac{1}{{T}_{{{{\rm{relax}}}}}}\left(\frac{\triangle {I}_{0}}{{I}_{0}}+\frac{\triangle {I}_{{\nu }_{{{{\rm{CPMG}}}}}}}{{I}_{{\nu }_{{{{\rm{CPMG}}}}}}}\right).$$

The data points of ${R}_{2}^{{{{\rm{eff}}}}}$ versus *ν*_CPMG_ were fitted to the 2-site CPMG model presented in ref. ^46^. We used the nonlinear least square method implemented by the trust-region algorithm. The model is as follows:

5 $$	{R}_{2,{{{\rm{fit}}}}}^{{{{\rm{eff}}}}}= {R}_{2}^{0}+\frac{{k}_{{{{\rm{ex}}}}}}{2}-{\nu }_{{{{\rm{CPMG}}}}}{\cosh }^{-1}\left({D}_{+}{\cosh }^{-1}\left({\eta }_{+}\right)-{D}_{-}\cos \left({\eta }_{-}\right)\right)\\ 	-\frac{1}{{T}_{{{{\rm{relax}}}}}}{{\mathrm{ln}}}\left({{\mathrm{Re}}}\left(1-{m}_{{{{\rm{D}}}}+}^{2}-{m}_{{{{\rm{Z}}}}+}^{2}+{m}_{{{{\rm{D}}}}+}{m}_{{{{\rm{Z}}}}+}+\frac{{m}_{{{{\rm{D}}}}+}+{m}_{{{{\rm{Z}}}}+}}{2}\sqrt{\frac{{p}_{{{{\rm{B}}}}}}{{p}_{{{{\rm{A}}}}}}}\right)\right)$$

where

6 $$\begin{array}{c}{D}_{\pm }=\frac{1}{2}\left(\pm 1+\frac{\psi +2{\Delta \omega }^{2}}{\sqrt{{\psi }^{2}+{\zeta }^{2}}}\right)\\ {\eta }_{\pm }={2}^{\frac{-3}{2}}\frac{1}{{\nu }_{{{{\rm{CPMG}}}}}}{\left(\pm \psi +\sqrt{{\psi }^{2}+{\zeta }^{2}}\right)}^{\frac{1}{2}}\\ \begin{array}{c}\psi ={{k}_{{{{\rm{ex}}}}}}^{2}-{\Delta \omega }^{2}\\ \zeta =-2\Delta \omega {k}_{{{{\rm{ex}}}}}\left({p}_{{{{\rm{A}}}}}-{p}_{{{{\rm{B}}}}}\right)\\ \begin{array}{c}{m}_{{{{\rm{D}}}}+}=-\frac{i{k}_{{{{\rm{ex}}}}}\sqrt{{{p}_{{{{\rm{A}}}}}p}_{{{{\rm{B}}}}}}}{\left({{k}_{{{{\rm{ex}}}}}}^{2}+{\Delta \omega }^{2}\right)}\left(-\Delta \omega +i{k}_{{{{\rm{ex}}}}}+2\Delta \omega \frac{\sin \left(\frac{-\Delta \omega +i{k}_{{{{\rm{ex}}}}}}{4{\nu }_{{{{\rm{CPMG}}}}}}\right)}{\sin \left(\frac{i{k}_{{{{\rm{ex}}}}}}{2{\nu }_{{{{\rm{CPMG}}}}}}\right)}\right)\\ {m}_{{{{\rm{Z}}}}+}=\frac{i{k}_{{{{\rm{ex}}}}}\sqrt{{{p}_{{{{\rm{A}}}}}p}_{{{{\rm{B}}}}}}}{\left({{k}_{{{{\rm{ex}}}}}}^{2}+{\Delta \omega }^{2}\right)}\left(\Delta \omega -i{k}_{{{{\rm{ex}}}}}-2\varDelta \omega \frac{\sin \left(\frac{\Delta \omega -i{k}_{{{{\rm{ex}}}}}}{4{\nu }_{{{{\rm{CPMG}}}}}}\right)}{\sin \left(\frac{-i{k}_{{{{\rm{ex}}}}}}{2{\nu }_{{{{\rm{CPMG}}}}}}\right)}\right)\end{array}\end{array}\end{array}$$

The fit per residue yielded the basal transverse relaxation rate ${R}_{2}^{0}$ in s^−1^, the exchange rate constant *k*_ex_ in s^−1^ (*k*_ex_ = *k*_AB_ + *k*_BA_), the populations *p*_A_ and *p*_B_, where *p*_B_ = 1- *p*_A_ and *p*_A_ > *p*_B_, and the ^15^N chemical shift Δ*ω* = Δ*ω*_N_ in s^−1^, assuming that Δ*ω*_H_ = 0$.$ For the group (global) fits, the values of *k*_ex_ and *p*_A_ were forced to be the same for the subset of residues selected from the per-residue fit based on the similar *k*_ex_ values within certain domain substructures. Fitting was performed using computational scripts adapted from Singh et al.^58^.

#### Evaluation of the conformational exchange

The presence of conformational exchange (CE) was assessed based on the relationships between the experimental data and the data fitted with the 2-site CPMG model^46^ as follows,

7 $${{{\rm{CE}}}}=\left\{\begin{array}{cc}{{{\rm{Yes}}}}; & \left({R}_{2,{{{\rm{fit}}}}}^{{{{\rm{eff}}}}}\left({{{{\rm{\nu }}}}}_{{{{\rm{CPMG}}}}}^{{{{\rm{first}}}}}\right)-{R}_{2,{{{\rm{fit}}}}}^{{{{\rm{eff}}}}}\left({\nu }_{{{{\rm{CPMG}}}}}^{{{{\rm{last}}}}}\right)\right) > {\max }_{i}\left|{R}_{2}^{{{{\rm{eff}}}}}\left({\nu }_{{{{\rm{CPMG}}}}}^{i}\right)-{R}_{2,{{{\rm{fit}}}}}^{{{{\rm{eff}}}}}({\nu }_{{{{\rm{CPMG}}}}}^{i})\right|\\ {{{\rm{Visible}}}}; & \begin{array}{c}\left({R}_{2,{{{\rm{fit}}}}}^{{{{\rm{eff}}}}}\left({\nu }_{{{{\rm{CPMG}}}}}^{{{{\rm{first}}}}}\right)-{R}_{2,{{{\rm{fit}}}}}^{{{{\rm{eff}}}}}\left({\nu }_{{{{\rm{CPMG}}}}}^{{{{\rm{last}}}}}\right)\right)\le {\max }_{i}\left|{R}_{2}^{{{{\rm{eff}}}}}\left({\nu }_{{{{\rm{CPMG}}}}}^{i}\right)-{R}_{2,{{{\rm{fit}}}}}^{{{{\rm{eff}}}}}\left({\nu }_{{{{\rm{CPMG}}}}}^{i}\right)\right|{{{\rm{and}}}}\\ \left({R}_{2,{{{\rm{fit}}}}}^{{{{\rm{eff}}}}}\left({\nu }_{{{{\rm{CPMG}}}}}^{{{{\rm{first}}}}}\right)-{R}_{2,{{{\rm{fit}}}}}^{{{{\rm{eff}}}}}\left({\nu }_{{{{\rm{CPMG}}}}}^{{{{\rm{last}}}}}\right)\right) > {{{\rm{RMSE}}}}\end{array}\\ {{{\rm{No}}}}; & \begin{array}{c}\left({R}_{2,{{{\rm{fit}}}}}^{{{{\rm{eff}}}}}\left({\nu }_{{{{\rm{CPMG}}}}}^{{{{\rm{first}}}}}\right)-{R}_{2,{{{\rm{fit}}}}}^{{{{\rm{eff}}}}}\left({\nu }_{{{{\rm{CPMG}}}}}^{{{{\rm{last}}}}}\right)\right)\le {{{\rm{RMSE}}}}\end{array}\end{array}\right.$$

where the index *i* ranges from the first to the last non-zero *ν*_CPMG_ (excluding the repeated data point) and RMSE corresponds to the root-mean-square error of the fit.

The reliability of the conformational exchange (RE) classification was estimated based on the relation of the repeated data point, ${R}_{2}^{{{{\rm{eff}}}}}$ (${\nu }_{{{{\rm{CPMG}}}}}^{{{{\rm{repeat}}}}}$), to the CPMG fit, ${R}_{2,{{{\rm{fit}}}}}^{{{{\rm{eff}}}}}$, in the following way,

8 $${{\rm{RE}}}=\left\{\begin{array}{cc}{{\rm{Reliable}}}; \hfill& \begin{array}{ccc}{{\rm{CE}}}={\mbox{Yes}}\, \hfill& {{\rm{and}}} & \left|{R}_{2}^{{{\rm{eff}}}}\left({\nu }_{{{\rm{CPMG}}}}^{{{\rm{repeat}}}}\right)-{R}_{2,{{\rm{fit}}}}^{{{\rm{eff}}}}\left({\nu }_{{{\rm{CPMG}}}}^{{{\rm{repeat}}}}\right)\right| < \left({R}_{2,{{\rm{fit}}}}^{{{\rm{eff}}}}\left({\nu }_{{{\rm{CPMG}}}}^{{{\rm{first}}}}\right)-{R}_{2,{{\rm{fit}}}}^{{{\rm{eff}}}}\left({\nu }_{{{\rm{CPMG}}}}^{{{\rm{last}}}}\right)\right)\, {{\rm{or}}}\\ {{\rm{CE}}}={\mbox{Visible}} \hfill& {{\rm{and}}} & \left|{R}_{2}^{{{\rm{eff}}}}\left({\nu }_{{{\rm{CPMG}}}}^{{{\rm{repeat}}}}\right)-{R}_{2,{{\rm{fit}}}}^{{{\rm{eff}}}}\left({\nu }_{{{\rm{CPMG}}}}^{{{\rm{repeat}}}}\right)\right| < {{\rm{RMSE\; or}}}\hfill\\ {{\rm{CE}}}={\mbox{No}} \hfill& {{\rm{and}}} & \left|{R}_{2}^{{{\rm{eff}}}}\left({\nu }_{{{\rm{CPMG}}}}^{{{\rm{repeat}}}}\right)-{R}_{2,{{\rm{fit}}}}^{{{\rm{eff}}}}\left({\nu }_{{{\rm{CPMG}}}}^{{{\rm{repeat}}}}\right)\right| < {\max }_{i}\left|{R}_{2}^{{{\rm{eff}}}}\left({\nu }_{{{\rm{CPMG}}}}^{i}\right)-{R}_{2,{{\rm{fit}}}}^{{{\rm{eff}}}}({{{\rm{\nu }}}}_{{{\rm{CPMG}}}}^{i})\right|\end{array}\\ {{\rm{Less\; reliable}}}; & \, {{\rm{otherwise}}}\hfill\end{array}\right.$$

where the index *i* ranges from the first to the last non-zero *ν*_CPMG_ (excluding the repeated data point) and ${\nu }_{{{{\rm{CPMG}}}}}^{{{{\rm{repeat}}}}}$ denotes the CPMG frequency at which the CPMG experiment was repeated.

#### Principal component analysis (PCA)

The spectral densities *J*(0), *J*(0.87*ω*_H_) and *J*(*ω*_N_) were considered for the PCA as variables with the known values for the residues of the three MurD states. Due to different scales of the original variables, standardization of the data was performed (i.e., to have a mean of zero and a standard deviation of one) to ensure that all variables contribute equally to the analysis.

By setting the specific criteria for the principal component (PC) scores, we have identified five sets of residues exhibiting exclusive dynamics (residues presented with the colored circles in Fig. 3). The criteria are presented in Table 2. For PC1 and PC3 (which indicate a compensation effect), we extracted the residues whose PC scores stand out in the positive and negative PC directions, while for PC2, which shows no compensation effect, we extracted the residues whose PC2 scores stand out from the others in the positive PC direction.

### Molecular dynamics simulations

Molecular dynamics (MD) simulations of representing four MurD states (MurD_apo_, MurD_ATP_, and MurD_inh_ with and without ATP) were performed starting from the complexes presented in ref. ^18^ using the NAMD^59^ MD package and Charmm36 force field parameters^60^. Force field parameters for ligands were obtained by CGenFF^61^, while partial charges were additionally optimized by Gaussian16 (Gaussian Inc., W.C. Gaussian 16). Explicit solvent environments were generated as an aqueous solution of DMSO (molar ratio 100:3) with the addition of 0.11 M NaCl and 0.02 M MgCl_2_ using in-house software for random distribution of solvent molecules at a given density by considering periodic boundary conditions. For water, we used TIP3P model. All four systems were subjected to energy minimization, followed by a series of 10 ns equilibration MD simulations. Subsequently, a 2 μs production NPT simulation was performed for each system applying no position restraints. Each system consisted of ~70,000 atoms and was confined in an orthogonal 89 Å^3^ simulation box. The temperature was maintained at 300 K by the Nosé–Hoover thermostat and the pressure of 1 bar was kept constant by the Parrinello–Rahman barostat. Long range electrostatic interactions were calculated with the particle-mesh Ewald (PME) method. A cutoff of 1.0 nm for short-range electrostatic interactions and 1.4 nm for van der Waals interactions was used.

For the two selected pairs of MurD subdomains, we further analyzed the relative movements, namely the movement of the CTD domain versus NTD-CeD and the β-meander (β12-β13-β14) subdomain versus the rest. In this analysis, the dynamic fluctuations of selected protein subdomains were visualized by plotting 2D cloud diagrams showing the distance between the centers of masses (CM) of the selected subdomains and the relative orientation of the domains during the MD simulation. The latter was determined as the direction of the domain mass tensor axis with the lowest moment of inertia for each domain.

The motions per residue from MD simulations were analyzed via the autocorrelation function $C\left(\tau \right)$ for the standard order parameter depending on the second-order Legendre polynomial of the angle between the consecutive orientations of the residue N-H backbone vector ${{{\boldsymbol{\mu }}}}\left(t\right)$:

9 $$C\left(\tau \right)={\left\langle {P}_{2}\left({{{\boldsymbol{\mu }}}}\left(t\right)\cdot {{{\boldsymbol{\mu }}}}\left(t+\tau \right)\right)\right\rangle }_{t}$$

where ${P}_{2}$ is the second-order Legendre polynomial, and the angle brackets denote a time average over trajectory. The spectral density function describing the power of the residue motion at different frequencies *ω*, is the Fourier Transform of the correlation function $C\left(\tau \right)$^62^:

10 $$J\left(\omega \right)=\frac{1}{5}{\int }_{0}^{\infty }C\left(\tau \right)\cos \left(\omega \tau \right)d\tau .$$

The classification of the dynamics per residue was based on the relationships between the areas under the spectral density curve in the four frequency ranges. Namely,

11 $${w}_{j}=\frac{1}{({\omega }_{j2}-{\omega }_{j1})}{\int }_{{\omega }_{j1}}^{{\omega }_{j2}}J(\omega )d\omega ;j=1,2,3,4$$

where $[{\omega }_{11},{\omega }_{12}]=[0,\,0.04]{GHz}$, $[{\omega }_{21},{\omega }_{22}]=[0.04,\,1]{GHz}$, $[{\omega }_{31},{\omega }_{32}]=\left[1,\,25\right]{GHz}$ and $[{\omega }_{41},{\omega }_{42}]=[25,\infty ) {GHz}.$ We further calculated the average area under the spectral density curve in the four frequency ranges for each residue type $t$, i.e.,

12 $$\bar{{w}_{j}^{t}}=1/{N}_{t} {\sum}_{{Res}=1}^{{N}_{t}\,}{w}_{j,{Res}}^{t}{;j}=1,2,3,4$$

where ${N}_{t}$ is the number of the type $t$ residues, and determined the dynamical deviation of each residue of this type as the difference

13 $${d}_{j,{Res}}={w}_{j,{Res}}-\bar{{w}_{j}^{t}}{;j}=1,2,3,4.$$

The criteria established for these spectral density-based MD results for residue classification are shown in Table 3 along with the corresponding experimental outcomes with which they are compared. Categorization is performed for all residues in various frequency ranges while determining its dominating role based on the largest deviation from the average value for a given residue type within a given frequency range.

### Reporting summary

Further information on research design is available in the Nature Portfolio Reporting Summary linked to this article.

## Supplementary information

Supplementary Information

Description of Additional Supplementary Files

Supplementary Data 1

Supplementary Data 2

Supplementary Data 3

Supplementary Data 4

Supplementary Data 5

Supplementary Data 6

Supplementary Data 7

Supplementary Data 8

Supplementary Data 9

Supplementary Data 10

Reporting Summary

## Data Availability

^1^HN, ^15^N chemical shifts of the MurD apo and bound states to support the NMR spectra in Fig. 1b and Fig. 6b can be found in Supplementary Data 1. Numerical data to support the graphical representation of Fig. 2, insets a, c and d, can be found in Supplementary Data 2. Numerical data to support the graphical representation of Fig. 3, insets c, d and e, can be found in Supplementary Data 3. Numerical data to support the graphical representation of Fig. 5, insets i), ii) and iii), can be found in Supplementary Data 4. The initial and final configurations of the MD trajectories of the MurD_apo_ state can be found in Supplementary Data 5 and 6, respectively. The initial and final configurations of the MD trajectories of the MurD_ATP_ state can be found in Supplementary Data 7 and 8, respectively. The initial and final configurations of the MD trajectories of the MurD_inh_ state can be found in Supplementary Data 9 and 10, respectively. All relevant data supporting the findings of this study are available upon request from the corresponding authors.
