# Supplementary material for: Mechanistic insight into the dynamics of Mur ligase through a comprehensive timescale-specific approach
Source: Commun Chem. 2025 Sep 29;8:285. doi: 10.1038/s42004-025-01675-z (PMC12480691; doi:10.1038/s42004-025-01675-z)
Supplement: Supplementary file 1 — Supplementary Information [file 42004_2025_1675_MOESM1_ESM.pdf]

Supplementary Information

Mechanistic insight into the dynamics of Mur ligase through a comprehensive timescale-specific approach

Iza Ogris[1,2], Barbara Zupančič[1]*, Izidor Sosič[3], Franci Merzel[1], Simona Golič Grdadolnik[1]*

[1]*Laboratory for Molecular Structural Dynamics, Theory Department, National Institute of Chemistry, Hajdrihova ulica 19, 1001 Ljubljana, Slovenia*
[2]*Faculty of Medicine, University of Ljubljana, Vrazov trg 2, 1000 Ljubljana, Slovenia*
[3]*Faculty of Pharmacy, University of Ljubljana, Aškerčeva 7, 1000 Ljubljana, Slovenia*

*Corresponding Authors: Simona Golič Grdadolnik, Barbara Zupančič

Corresponding authors e-mail: simona.grdadolnik@ki.si; barbara.zupancic@ki.si

List of Supplementary Figures:

Figure S1: Experimental backbone relaxation parameters R_1, R_2 and hetNOE for $MurD_{apo}$ (in black), $MurD_{ACP}$ (in magenta) and $MurD_{inh}$ (in green). 3

Figure S2: R_1R_2, $J(0)$, $J(\omega_N)$ and $J(0.87\omega_H)$ for (**a**) $MurD_{apo}$ (in black) (**b**) $MurD_{ACP}$ (in magenta) and (**c**) $MurD_{inh}$ (in green). 4

Figure S3: ^{1}H-^{15}N TROSY spectrum of ^{2}H/^{15}N-labeled $MurD_{apo}$ 6

Figure S4: ^{1}H-^{15}N TROSY spectrum of ^{2}H/^{15}N-labeled MurD bound to AMP-PCP ($MurD_{ACP}$). 7

Figure S5: ^{1}H-^{15}N TROSY spectrum of ^{2}H/^{15}N-labeled MurD bound to inhibitor **1** ($MurD_{inh}$) 8

Figure S6: Chemical shift perturbation map for ^{2}H^{15}N MurD bound to (A) 8 equivalents of AMP-PCP and (B) 4 equivalents of inhibitor. 9

Figure S7: Bar diagram showing the number of residues assigned to each PCA group (G1-G5) in three states of MurD: $MurD_{apo}$, $MurD_{ACP}$ and $MurD_{inh}$. 10

Figure S8: Inter-domain distances between CM (center of mass) in all-atom MD simulations. 10

List of Supplementary Tables:

Table S1: Conformational analysis of MurD crystal structure (PDB 8VW2) based on RMSD comparison. 11

Table S2: Number of residues for which values were obtained for experimental or calculated parameters for 430 non-proline residues in the MurD protein. 11

Table S3: Overview of the site-specific comparison of local backbone dynamics**.** 12

Table S4: Number of segments with residues showing the dynamics of a particular group per domain for all three states of MurD. 18

Table S5: Overview of the segments and the residues exhibiting conformational exchange with k_{ex} values.. 19

Table S6: Comparison of prediction of conformational exchange dynamics on μs-ms time scale using PCA of reduced spectral densities 21

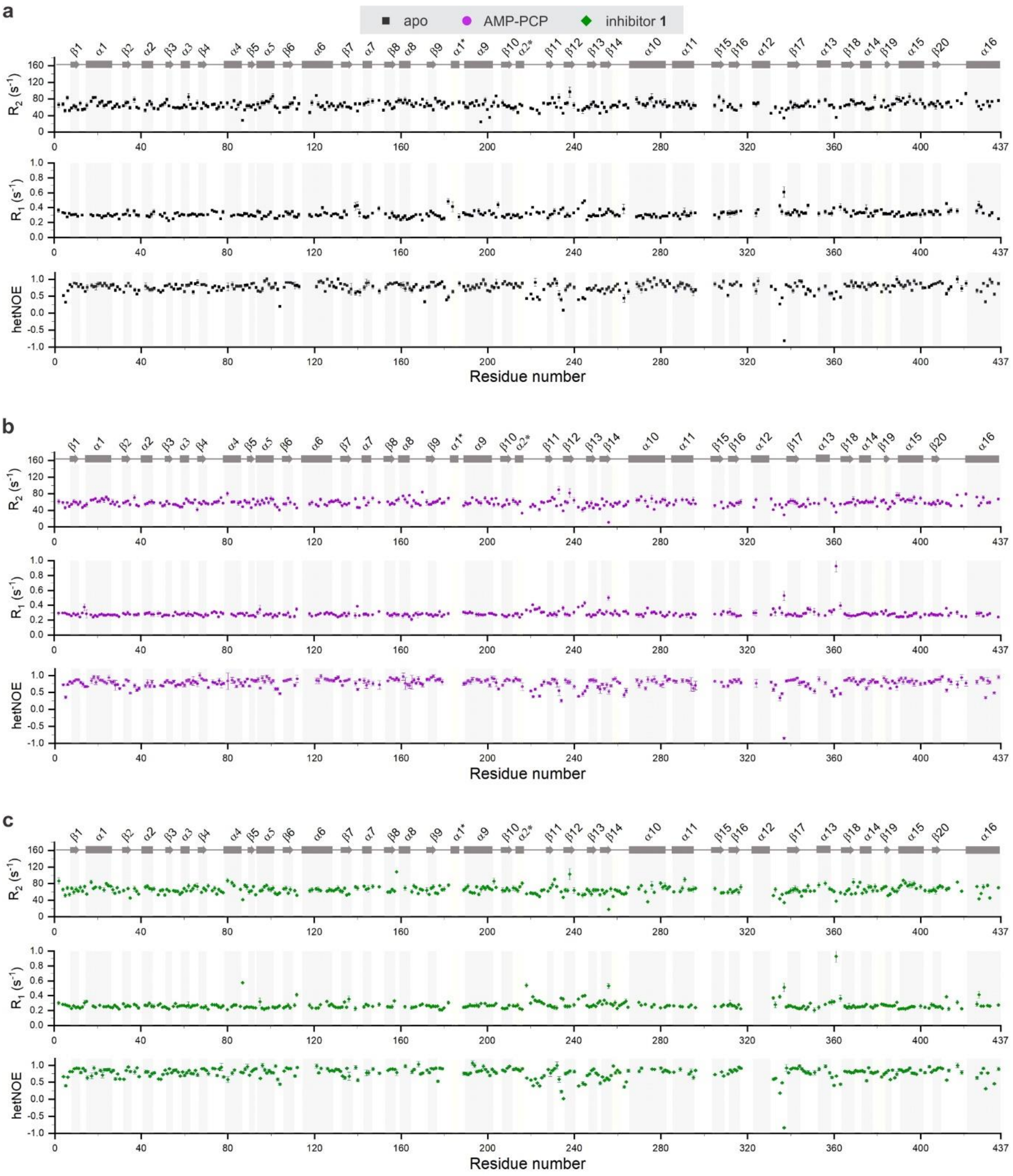

Figure S1: Experimental backbone relaxation parameters R_1, R_2 and hetNOE for MurD$_{apo}$ (in black), MurD$_{ACP}$ (in magenta) and MurD$_{inh}$ (in green) all taken at 800 MHz, 25 °C, with a protein concentration of 0.27 mM and four equivalents of ligands added. Grey areas represent residues in α-helixes or β-sheets labelled as described in Bertrand *et al.*[1]. Elements that are also observed in some X-ray structures, but not mentioned in Bertrand, are coloured in orange and/or marked with an asterisk. The estimation of the experimental errors in the R_1 and R_2 values was determined based on the deviation between duplicated spectra. The errors in the hetNOE values were determined based on the uncertainties of the peak intensities estimated from the noise level of the spectra, as described by Farrow *et al.*[2].

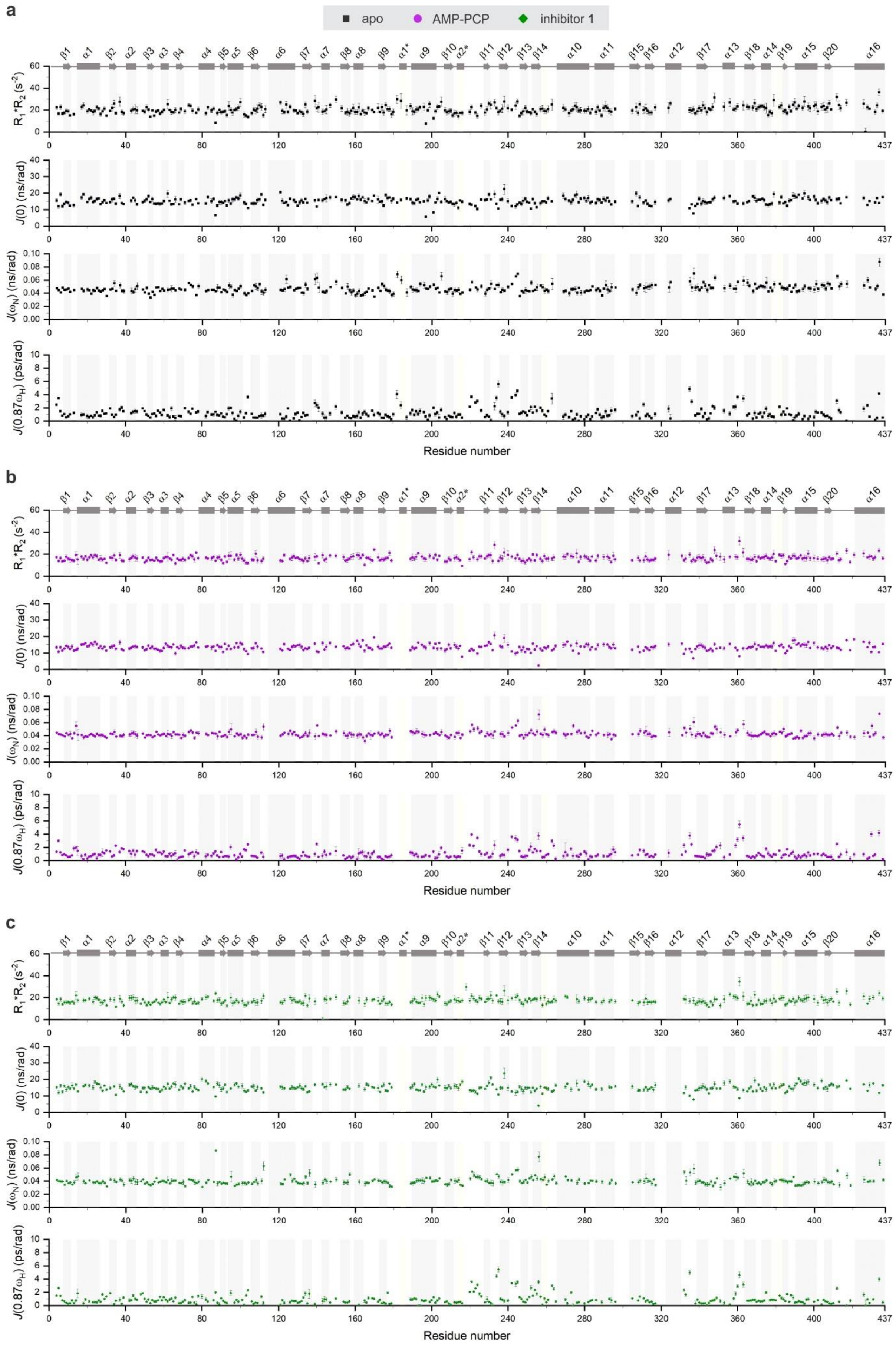
a
b
c
apo
AMP-PCP
inhibitor 1
β1 α1 β2 α2 β3 α3 β4 α4 β5 α5 β6 α6 β7 α7 β8 α8 β9 α1* α9 β10 α2* β11 β12 β13 β14 α10 α11 β15 β16 α12 β17 α13 β18 α14 β19 α15 β20 α16
R1*R2 (s-2)
J(0) (ns/rad)
J(ωN) (ns/rad)
J(0.87ωH) (ps/rad)
Residue number

Figure S2: $\boldsymbol{R_1R_2}$, $\boldsymbol{J(0)}$, $\boldsymbol{J(\omega_N)}$ and $\boldsymbol{J(0.87\omega_H)}$ for (a) MurD$_{apo}$ (in black) (b) MurD$_{ACP}$ (in magenta) and (c) MurD$_{inh}$ (in green). Secondary structure elements are presented and labeled as described by Bertrand *et al.*[1]. Helical turns that are observed in some X-ray structures, are marked with an asterisk. The errors in the R_1R_2, $J(0)$, $J(\omega_N)$ and $J(0.87\omega_H)$ values were estimated through error propagation.

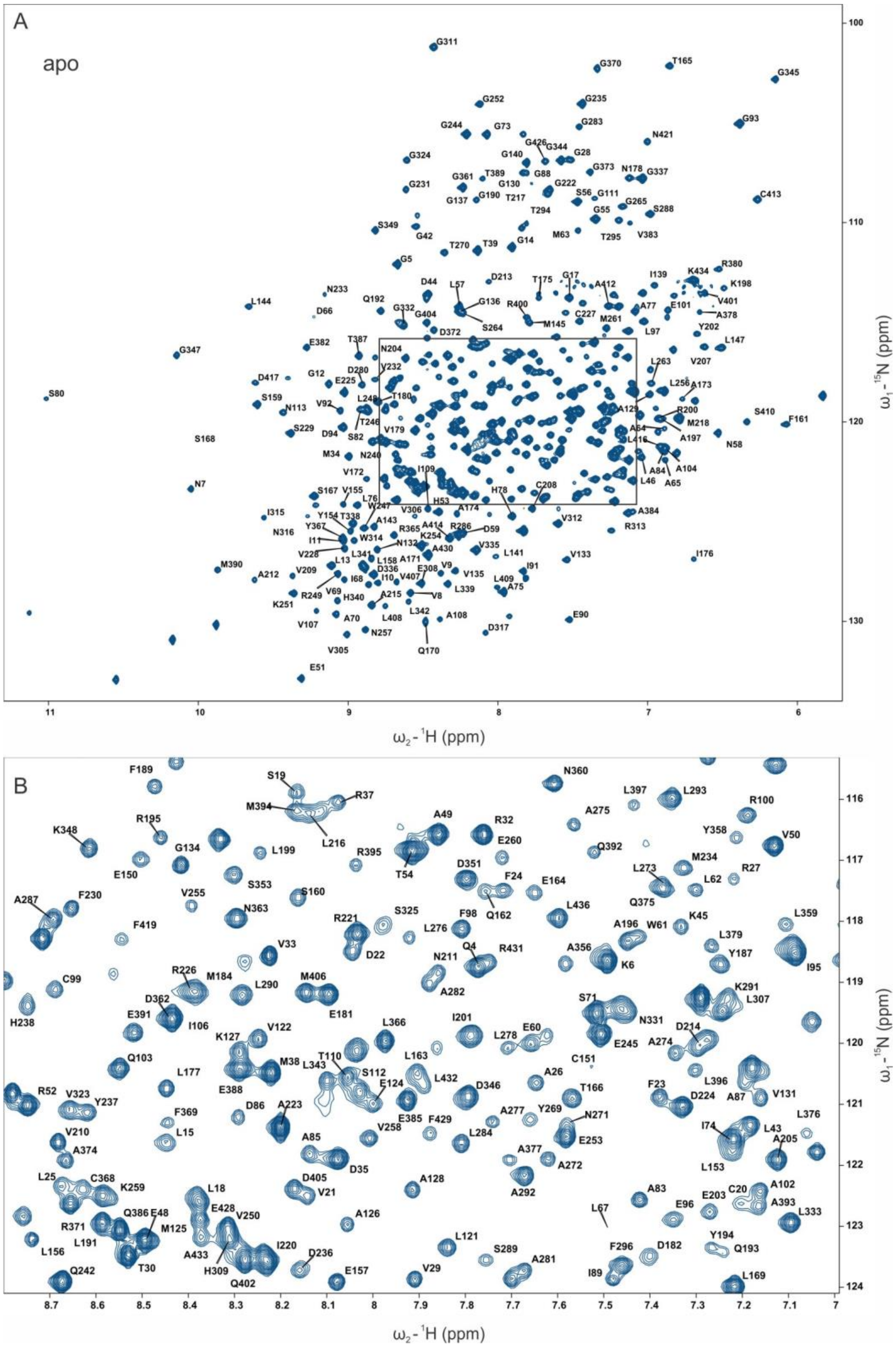

Figure S3: ^{1}H-^{15}N TROSY spectrum of ^{2}H/^{15}N-labeled MurD$_{apo}$ at 0.27 mM concentration in 20 mM Tris-d_6 (pH 7.2), 100 mM NaCl, 5 mM $MgCl_2$, 1 mM TCEP and 10% DMSO-d_6 at 25 °C, acquired on a 800 MHz spectrometer. Some assigned residues are labeled in (A) the full spectrum, while additional residues are labeled in (B) the magnified region for enhanced clarity.

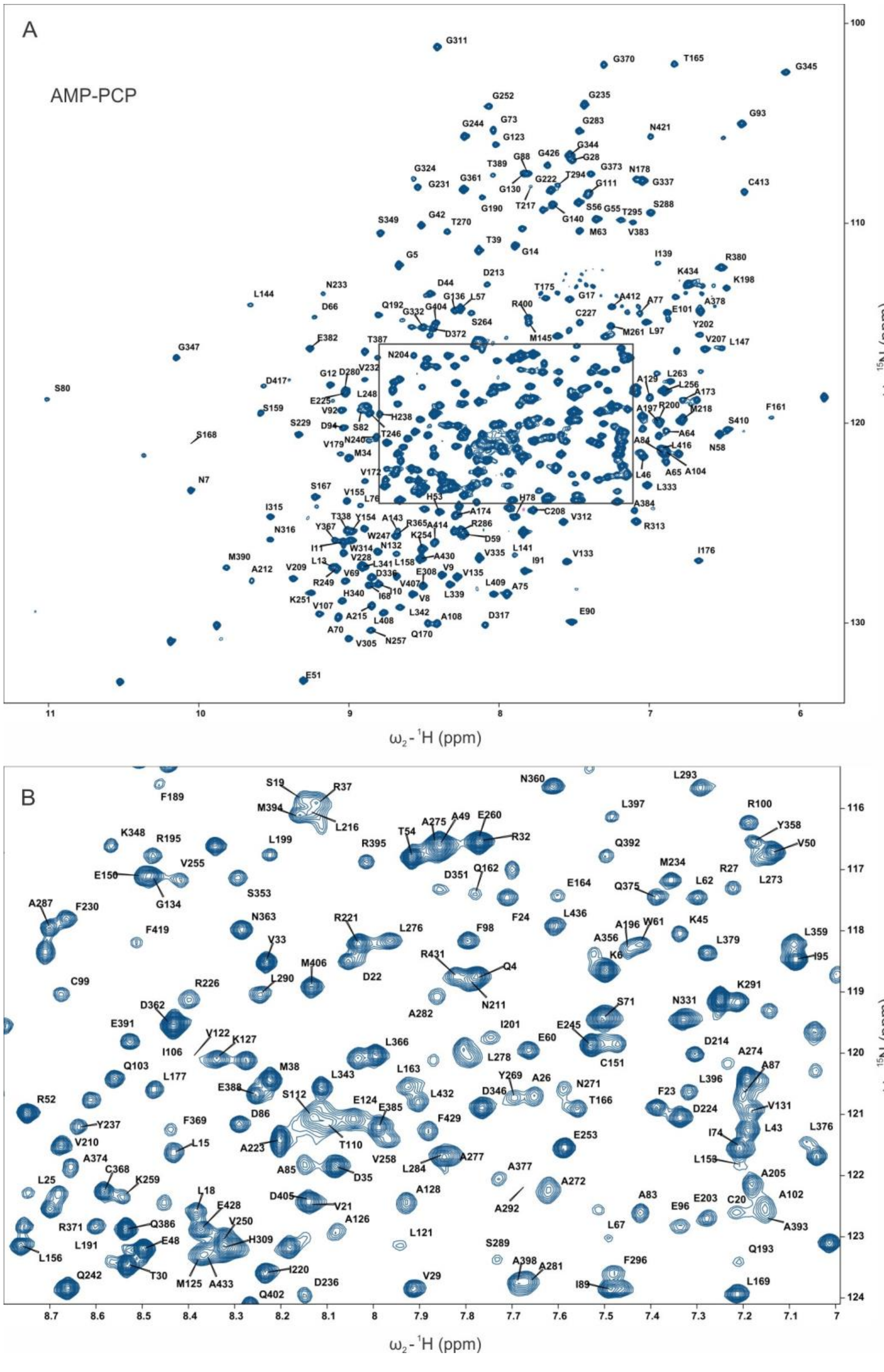

Figure S4: ^{1}H-^{15}N TROSY spectrum of ^{2}H/^{15}N-labeled MurD bound to AMP-PCP ($MurD_{ACP}$) in 20 mM Tris-d_6 (pH 7.2), 100 mM NaCl, 5 mM $MgCl_2$, 1 mM TCEP and 10% DMSO-d_6 at 25 °C, acquired on a 800 MHz spectrometer. MurD concentration is 0.27 mM and AMP-PCP is added in 8 equivalents. Some assigned residues are labeled in (A) the full spectrum, while additional residues are labeled in (B) the magnified region for enhanced clarity.

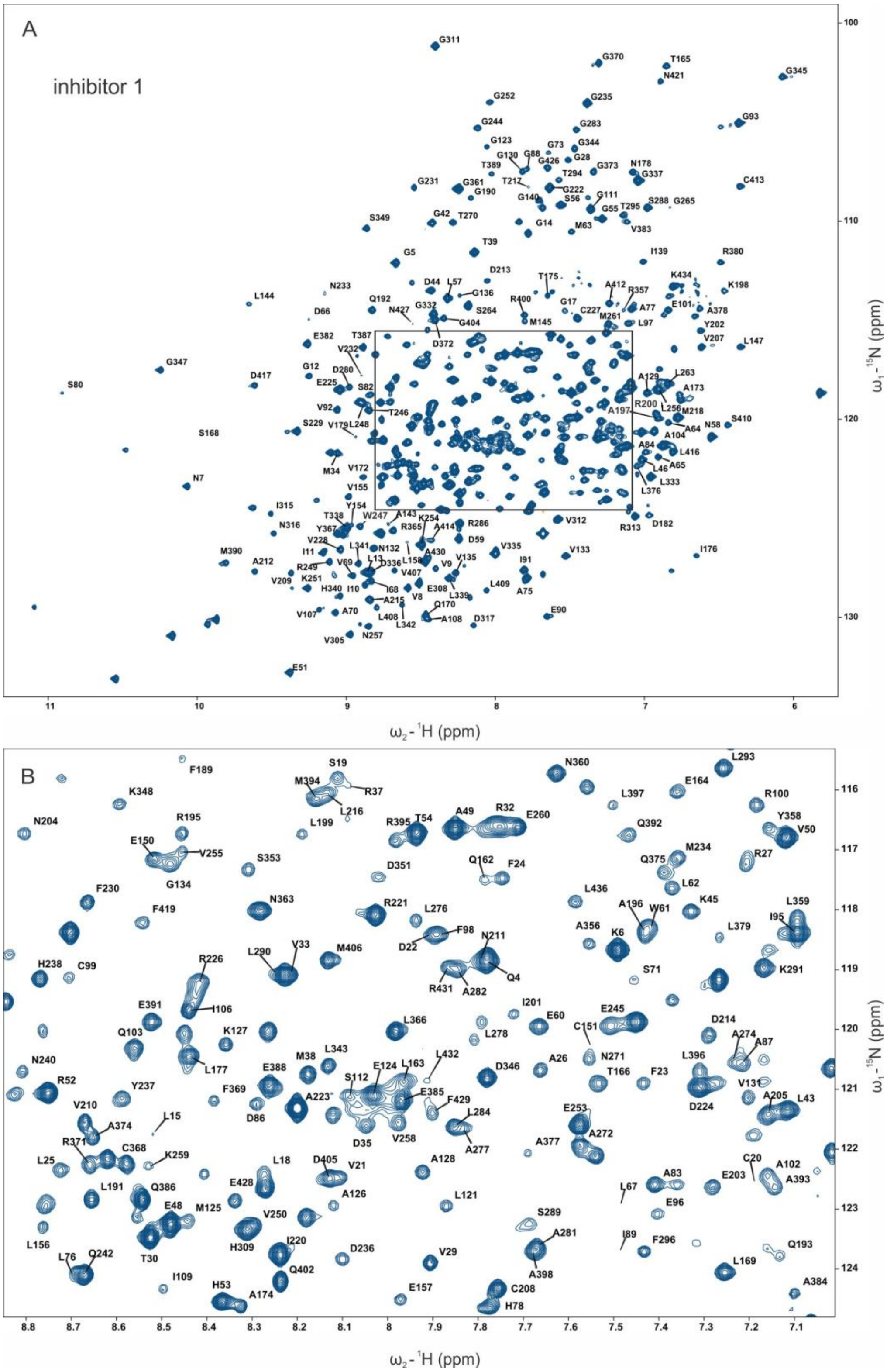

Figure S5: ^{1}H-^{15}N TROSY spectrum of $^{2}H/^{15}N$-labeled MurD bound to inhibitor 1 ($MurD_{inh}$) in 20 mM Tris-*d*6 (pH 7.2), 100 mM NaCl, 5 mM $MgCl_2$, 1 mM TCEP and 10% DMSO-d_6 at 25 °C, acquired on a 800 MHz spectrometer. MurD concentration is 0.27 mM and inhibitor is added in 4 equivalents. Some assigned residues are labeled in (A) the full spectrum, while additional residues are labeled in (B) the magnified region for enhanced clarity.

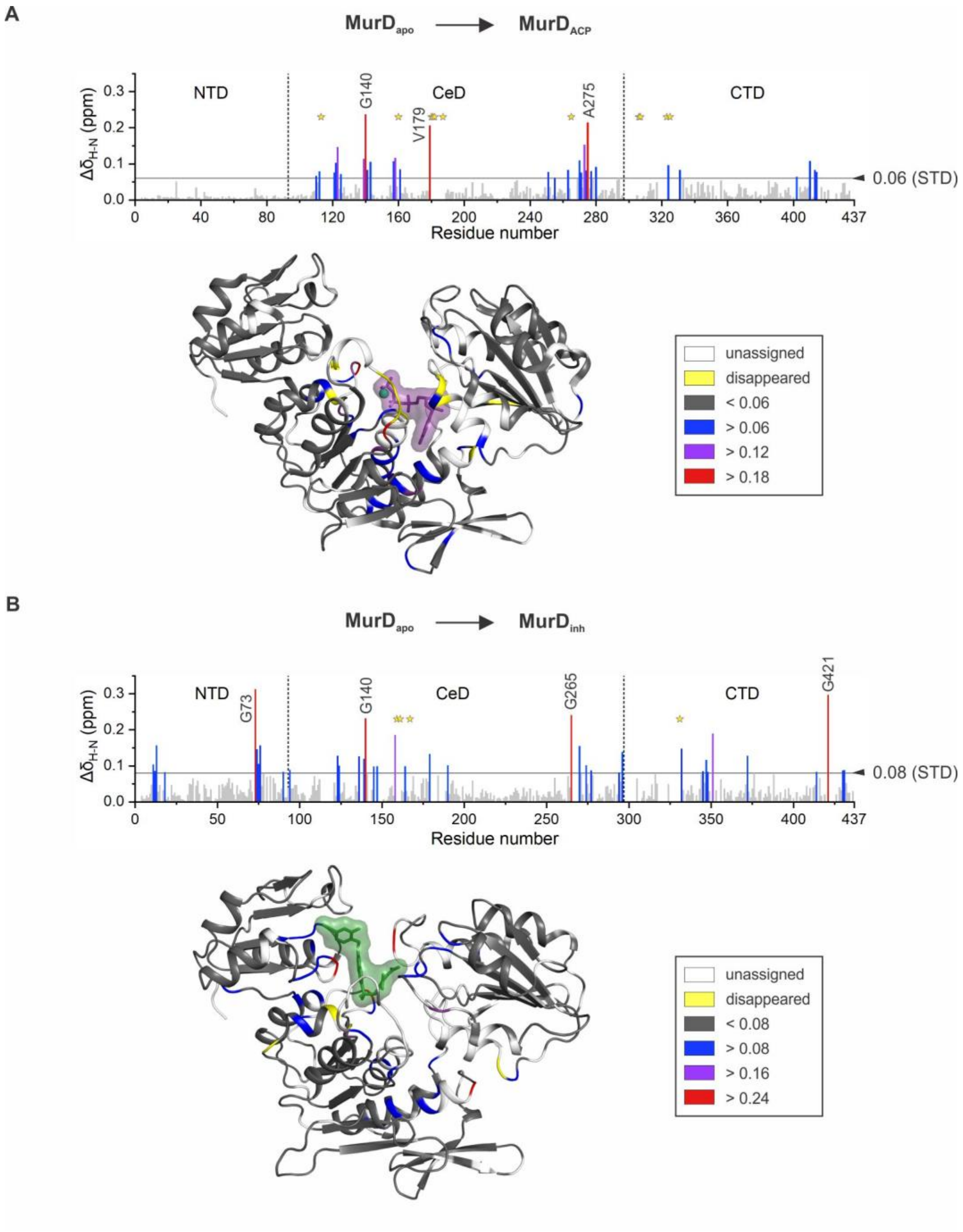

Figure S6: Chemical shift perturbation map for $^{2}H^{15}N$ MurD bound to (A) 8 equivalents of AMP-PCP and (B) 4 equivalents of inhibitor. Residues with values above the threshold are plotted to the X-ray structures (ATP-bound: PDB 8VW2[3], inhibitor-bound: PDB 2XPC[4]). Residues with values exceeding the threshold are shown in colors and mapped onto the corresponding structures. Threshold values, calculated as standard deviations, are 0.06 for AMP-PCP and 0.08 for the inhibitor. Residues are color-coded based on their values relative to the threshold and maximum: blue indicates values in the range of 1/3 above the threshold, violet indicates 2/3 above the threshold, and red represents 3/3 above the threshold. Residues with the strongest CSPs (in red) are labeled. Residues that disappeared during the titration are marked with yellow stars on the CSP plot and presented in yellow on the X-ray structure.

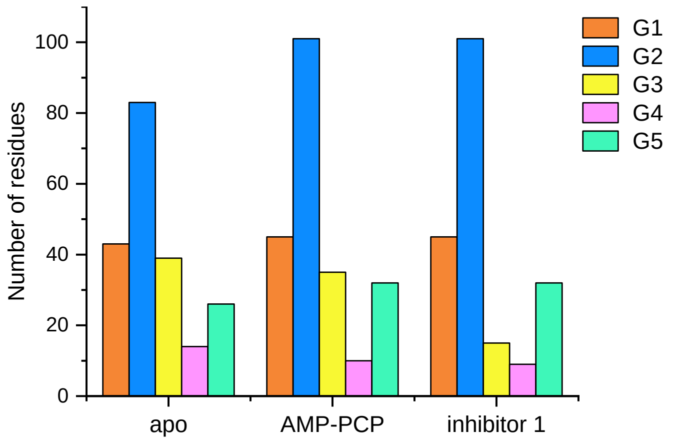

Figure S7: Bar diagram showing the number of residues assigned to each PCA group (G1-G5) in three states of MurD: $MurD_{apo}$, $MurD_{ACP}$ and $MurD_{inh}$.

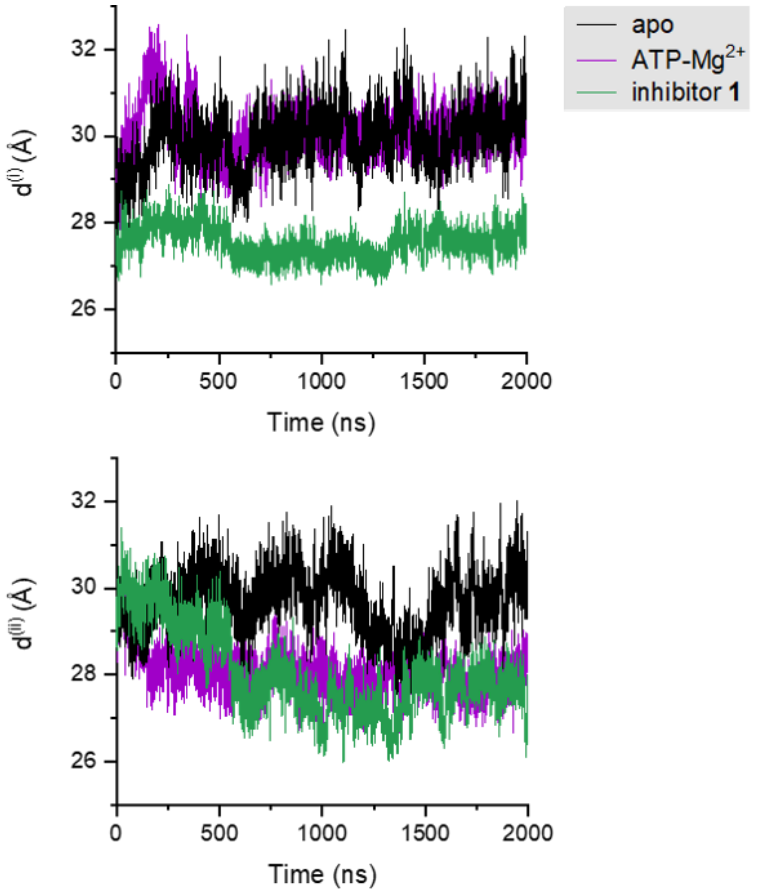

Figure S8: Inter-domain distances between CM (center of mass) of: (above) CTD versus NTD-CeD, $d^{(i)}$, and (bellow) β-meander versus the rest of the protein, $d^{(ii)}$, as a function of time in all-atom MD simulations.

Table S1: Analysis of conformation of MurD crystal structure (PDB 8VW2) based on RMSD comparison.

	apo<br>PDB **5A5E**[5]	UMA+ADP-bound<br>PDB **5A5F**[5]	UMA+ADP-bound<br>PDB **2UAG**[6]	apo<br>PDB **1E0D**[1]
ATP-bound<br>PDB **8VW2**	0.293 Å	0.840 Å	1.371 Å	3.162 Å
conformation	*semi-closed*	*semi-closed*	*closed*	*open*

Table S2: Number of residues for which values were obtained for experimental or calculated parameters for 430 non-proline residues in the MurD protein.

**Experimental or calculated parameters**	**Number of residues with obtained values**		
	**$MurD_{apo}$**	**$MurD_{ACP}$**	**$MurD_{inh}$**
$J(0)$, $J(\omega_N)$, $J(0.87\omega_H)$	302	314	287
R_2	343	327	313
R_1	311	331	302
hetNOE	349	328	313
Assigned residues	356	340	336

Table S3: Overview of the site-specific comparison of local backbone dynamics. Residues of $MurD_{apo}$, $MurD_{ACP}$, and $MurD_{inh}$ with specific dynamic properties are labeled in black, purple, and green, respectively. The secondary structure annotations are taken from Bertrand *et al.*[1]. Explanation of the groups G1-G5 is as follows: G1 - residues showing decreased $J(0)$ compensating for the increased $J(0.87\omega_H)$ and $J(\omega_N)$; G2 - residues showing increased $J(0)$ compensating for the decreased $J(0.87\omega_H)$ and $J(\omega_N)$ G3: Residues showing increased $J(0)$ and $J(\omega_N)$, without compensation of $J(0.87\omega_H)$ G4: Residues showing decreased $J(\omega_N)$ dynamics compensating for the increased $J(0.87\omega_H)$ G5: Residues showing increased $J(\omega_N)$ compensating for the decreased $J(0.87\omega_H)$. Note that a 'Yes' in the column 'Indication of conformational exchange dynamics from PC results' is based on the presence of G3 residues in this particular segment and means that intermediate conformational exchange dynamics is expected for this segment. A 'No' means that this is not the case.

Segment = Secondary structure element	*Sequence*	G1	G2	G3	G4[#]	G5[#]	*Indication of conformational exchange dynamics from PC results*	*Residues with conformational exchange dynamics determined by CPMG RD method*	*Residues with increased R_1R_2 values*	*Residues with R_{ex} using approach of Kneller et al.*[7]
NTD										
N-term	1-7	5<br>4,5<br>5	-<br>-<br>-	-<br>-<br>-	4<br>6<br>4,6	-<br>-<br>-	No<br>No<br>No	-<br>-<br>-	-<br>-<br>-	-<br>-<br>-
β1<br>**interaction with inhibitor* ***1***	8-11<br>11	-<br>-<br>-	-<br>10<br>8,10	-<br>-<br>-	-<br>-<br>-	10<br>-<br>9	No<br>No<br>No	-<br>-<br>-	-<br>-<br>-	-<br>-<br>-
β1-α1<br>**interaction with inhibitor* ***1***	12-14<br>12	-<br>13,14<br>-	-<br>12<br>12	-<br>-<br>14	-<br>-<br>-	-<br>-<br>13	No*<br>No<br>Yes	-<br>-<br>12	-<br>14<br>14	-<br>-<br>-
α1	15-26	-<br>-<br>15	18,21,22,24,26<br>17-25<br>18,19,21,23-26	25<br>-<br>-	-<br>-<br>-	-<br>-<br>-	Yes<br>No<br>No	-<br>-<br>-	18<br>22,24<br>24	18<br>24<br>24
α1-β2	27-31	-<br>-<br>28	29<br>27,29<br>27,29	-<br>-<br>-	-<br>-<br>-	-<br>-<br>-	No<br>No<br>No	-<br>-<br>-	-<br>-<br>-	-<br>-<br>-
β2<br>**interaction with inhibitor* ***1***	32-35<br>34,35	-<br>-<br>32	33<br>33<br>34	34<br>34<br>-	32<br>35<br>-	-<br>-<br>-	Yes<br>Yes<br>No	34<br>-<br>34	34<br>-<br>-	-<br>-<br>-
β2-α2<br>**interaction with inhibitor* ***1***	36-40<br>36,37	-<br>-<br>-	-<br>-<br>-	37<br>-<br>37	-<br>-<br>-	-<br>-<br>38	Yes<br>No<br>Yes	37, 39<br>-<br>-	37<br>37<br>-	-<br>37<br>-
α2	41-45	-<br>-<br>-	-<br>45<br>45	-<br>44<br>-	45<br>-<br>-	-<br>-<br>-	No<br>Yes<br>No	42<br>43<br>43, 44	-<br>-<br>44	-<br>-<br>-
α2-β3	46-51	46<br>-<br>48,49	-<br>-<br>46	51<br>-<br>-	49,50<br>46<br>-	-<br>-<br>-	Yes<br>No<br>No	-<br>-<br>-	-<br>-<br>-	-<br>-<br>-

β3	52-54	-<br>-<br>-	52,54<br>52,54<br>52,54	-<br>-<br>-	-<br>-<br>-	-<br>-<br>-	No<br>No<br>No	-<br>-<br>-	-<br>-<br>-	-<br>-<br>-
β3-α3<br>**interaction with inhibitor* ***1***	55-58<br>57	57<br>56<br>-	-<br>-<br>-	-<br>-<br>-	-<br>-<br>-	56<br>-<br>56	No<br>No<br>No	-<br>-<br>-	-<br>-<br>-	-<br>-<br>-
α3	59-62	-<br>60<br>-	-<br>61,62<br>-	-<br>-<br>-	60<br>-<br>60	-<br>-<br>-	No<br>No<br>No	-<br>-<br>-	62<br>-<br>62	62<br>-<br>-
α3-β4	63-66	64<br>-<br>-	63,66<br>63,64<br>65	-<br>65<br>-	-<br>-<br>-	-<br>-<br>-	No<br>Yes<br>No	-<br>-<br>-	-<br>65<br>65,66	-<br>-<br>-
β4	67-70	-<br>-<br>-	69<br>69<br>69	-<br>-<br>-	-<br>-<br>-	-<br>-<br>-	No*<br>No<br>No	-<br>-<br>-	-<br>-<br>-	-<br>-<br>-
β4-α4<br>**interaction with inhibitor* ***1***	71-78<br>71-74	75,78<br>78<br>-	-<br>76<br>71,76	74<br>74,75,77<br>-	-<br>71<br>-	-<br>73<br>73	Yes<br>Yes<br>No	77<br>73<br>-	73<br>74,77<br>-	-<br>-<br>-
α4	79-86	-<br>-<br>-	85,86<br>82,83<br>82,86	-<br>86<br>-	-<br>-<br>-	-<br>85<br>-	No<br>Yes<br>No*	-<br>-<br>85	-<br>-<br>82,83	-<br>-<br>82
α4-β5	87-89	-<br>-<br>87	-<br>88<br>88,89	-<br>-<br>-	-<br>-<br>-	89<br>89<br>-	No<br>No<br>No	-<br>-<br>-	-<br>-<br>87	-<br>-<br>-
β5	90-92	-<br>-<br>-	92<br>92<br>92	-<br>-<br>-	-<br>90<br>-	-<br>-<br>-	No<br>No<br>No	-<br>-<br>-	-<br>-<br>-	-<br>-<br>-
β5-α5	93	-<br>-<br>-	-<br>-<br>-	-<br>-<br>-	-<br>-<br>-	-<br>-<br>-	No<br>No<br>No	-<br>-<br>-	-<br>-<br>-	-<br>-<br>-
CeD										
α5	94-101	94,95<br>95<br>95	96-99<br>97-99,101<br>97-99,101	100<br>100<br>-	-<br>-<br>-	-<br>94<br>-	Yes<br>Yes<br>No	-<br>-<br>94^{V},101	100<br>98,100<br>-	-<br>-<br>-
α5-β6	102-105	-<br>104<br>-	-<br>-<br>-	-<br>-<br>-	-<br>-	-<br>-<br>102	No<br>No<br>No	-<br>-<br>-	-<br>-<br>-	-<br>-<br>-
β6	106-110	-<br>-<br>-	109<br>109<br>106,107,109	110<br>108<br>-	-<br>-<br>-	107,108<br>-<br>108	Yes<br>Yes<br>No	-<br>-<br>-	110<br>108<br>-	-<br>-<br>-
β6-α6<br>**interaction with ATP-*Mg^{2+}	111-114<br>111-114	-<br>-<br>-	111,113<br>-<br>-	-<br>-<br>-	-<br>-<br>-	-<br>112<br>-	No<br>No*<br>No*	-<br>111, 112<br>-	-<br>-<br>112	-<br>-<br>-
α6<br>**interaction with ATP-*Mg^{2+}	115-128<br>115-117<br>115	125<br>-<br>-	122,128<br>127<br>121,122,127	-<br>128<br>-	-<br>-<br>-	124<br>126<br>123,126,128	No<br>Yes<br>No	-<br>121, 126^{V}<br>-	121,124<br>123<br>126	121<br>-<br>-

**interaction with inhibitor **1***										
α6-β7	129-132	-<br>-<br>-	132<br>129-132<br>130,131	-<br>-<br>-	-<br>-<br>-	131<br>-<br>129	No<br>No<br>No	-<br>130<br>-	-<br>-<br>-	-<br>-<br>-
β7	133-137	-<br>-<br>136	-<br>-<br>-	134<br>-<br>-	-<br>-<br>-	-<br>134,136<br>-	Yes<br>No<br>No	133, 136<br>136<br>135	134<br>-<br>-	-<br>-<br>-
β7-α7<br>***rearranges with UMA binding**<br>**interaction with inhibitor **1***	138-142<br>138,139	139,140<br>140<br>-	-<br>139<br>139	-<br>-<br>-	-<br>-<br>-	-<br>-<br>-	No<br>No<br>No*	139, 140, 141<br>139, 140, 141<br>-	139<br>-<br>-	-<br>140<br>-
α7	143-146	-<br>-<br>-	143-145<br>145<br>145	-<br>143<br>144	-<br>-<br>-	-<br>-<br>-	No<br>Yes<br>Yes	-<br>144^{V}<br>-	-<br>143<br>-	-<br>-<br>-
α7-β8	147-152	-<br>-<br>-	-<br>-<br>147	147,150<br>150<br>150	-<br>-<br>-	-<br>-<br>-	Yes*<br>Yes*<br>Yes*	147<br>-<br>-	147,150<br>147<br>150	-<br>147<br>-
β8<br>**interaction with ATP-Mg^{2+}*	153-157<br>157	-<br>-<br>-	155<br>156<br>156	153<br>-<br>-	-<br>-<br>-	156<br>157<br>-	Yes<br>No<br>No	157<br>157<br>-	-<br>-<br>157	-<br>-<br>-
β8-α8<br>**interaction with inhibitor **1***	158-159<br>159	-<br>-<br>-	158,159<br>158,159<br>-	-<br>-<br>-	-<br>-<br>-	-<br>-<br>-	No<br>No<br>-**	158<br>158, 159<br>-	-<br>-<br>-	-<br>-<br>-
α8<br>**interaction with inhibitor **1***	160-164<br>161	-<br>-<br>-	160,161,163,164<br>161<br>162	-<br>162<br>-	-<br>163<br>-	-<br>-<br>-	No<br>Yes<br>No*	-<br>-<br>163	-<br>161,164<br>-	-<br>161,164<br>-
α8-β9	165-172	-<br>167<br>-	167,170<br>166<br>166	172<br>170<br>-	169<br>-<br>169	-<br>172<br>172	Yes<br>Yes<br>No	166, 170<br>167^{V}<br>-	-<br>170<br>-	-<br>170<br>-
β9	173-176	-<br>-<br>-	-<br>175,176<br>174,175	-<br>-<br>-	-<br>-<br>-	174,175<br>174<br>176	No<br>No<br>No	-<br>-<br>-	176<br>-<br>-	176<br>-<br>-
loop β9-α1-α9*<br>***unresolved in few X-ray structures***<br>**interaction with ATP-Mg^{2+}*<br>**interaction with inhibitor **1***	177-189<br>178<br>182,183,<br>186	182<br>189<br>-	177-180,187<br>177<br>178-179	189<br>178	-<br>-<br>-	-<br>-<br>-	Yes<br>Yes*<br>No*	181, 182, 184<br>179^{V}, 189<br>189	182,184<br>178	-<br>-<br>-
α9	190-202	194,196,201<br>190,201<br>-	193,198<br>195,196,199<br>190,200	199<br>193<br>197,199	192<br>-<br>201	-<br>191,192<br>193,194	Yes<br>Yes<br>Yes	193<br>193, 198<br>190, 202	199<br>193,202<br>199	-<br>-<br>-
α9-β10	203-206	-<br>205<br>-	204<br>204<br>-	203<br>-<br>204	-<br>-<br>-	-<br>-<br>-	Yes<br>No<br>Yes*	205<br>-<br>-	205<br>-<br>203,204	-<br>-<br>203
β10	207-211	-	207,208	-	-	210	No	-	-	-

		-<br>-	209<br>207,209	-<br>-	-<br>-	-<br>210	No<br>No	-<br>-	-<br>-	-<br>-
β10-α2-β11*<br>***(unresolved in X-ray structures)***	212-227<br>(221-225)	221,223<br>220-222,224<br>220-226	215,216<br>212,215<br>212,213,215,216	226<br>225<br>-	-<br>-<br>-	-<br>213,214,226<br>214	Yes<br>Yes<br>No	220<br>226<br>222, 227^V	-<br>-<br>-	-<br>-<br>-
β11	228-230	-<br>-<br>-	228,229<br>228,230<br>230	230<br>229<br>-	-<br>-<br>-	-<br>-<br>228	Yes<br>Yes<br>No	228<br>228<br>-	230<br>230<br>-	230<br>230<br>-
β11-β12	231-236	234,235<br>-<br>234,235	-<br>231,236<br>231,232	233<br>233<br>-	236<br>-<br>-	231<br>-<br>-	Yes<br>Yes<br>No	231, 234<br>233, 236<br>-	231,233<br>231<br>231	-<br>-<br>231
β12	237-240	-<br>-<br>-	237<br>237<br>-	-<br>240<br>-	-<br>-<br>-	-<br>-<br>240	No<br>Yes<br>No	237, 238, 240<br>238, 240<br>240	238<br>238,240<br>238	238<br>238<br>238
β12-β13<br>***(unresolved in X-ray structures)***	241-246	242,244,245<br>242,244,245<br>242,244,245	-<br>-<br>-	-<br>-<br>-	-<br>246<br>246	-<br>-<br>-	No<br>No<br>No	-<br>-<br>244^V	245<br>-<br>-	-<br>-<br>-
β13	247-250	-<br>-<br>-	-<br>248<br>-	-<br>-<br>-	-<br>-<br>-	-<br>-<br>249	No<br>No<br>No	249, 250<br>-<br>-	-<br>-<br>-	-<br>-<br>-
β13-β14	251-252	252<br>251,252<br>251,252	-<br>-<br>-	-<br>-<br>-	-<br>-<br>-	-<br>-<br>-	No<br>No<br>No	251<br>-<br>-	-<br>-<br>-	-<br>-<br>-
β14	253-257	257<br>256,257<br>253-256	253<br>-<br>-	-<br>253,255<br>-	-<br>-<br>-	-<br>-<br>-	No<br>Yes<br>No	255<br>255<br>253, 257	-<br>253,255<br>-	-<br>-<br>-
β14-α10	258-265	258,263<br>263,264<br>263,264	259<br>261<br>265	-<br>-<br>259	261<br>-<br>-	-<br>259<br>258	No<br>No<br>Yes	263, 264<br>258<br>264	263<br>-<br>-	-<br>-<br>-
α10<br>**interaction with ATP-Mg^{2+}*	266-282<br>267,268,<br>270,271	-<br>-<br>-	269,270,272,<br>274-276, 278,<br>280-282<br>269,281,282<br>271,276,280,282	-<br>271,276,278,<br>280<br>270	-<br>273<br>-	277<br>274<br>277	No<br>Yes<br>Yes*	-<br>269<br>272^V, 281^V, 282^V	-<br>271,276<br>270,271,274	275<br>271<br>-
α10-α11	283-285	-<br>-<br>-	-<br>-<br>283,284	-<br>-<br>-	-<br>-<br>-	284<br>284<br>-	No<br>No<br>No	-<br>283^V<br>-	-<br>-<br>-	-<br>-<br>-
α11	286-295	294<br>-<br>-	288,290,292,293<br>290,292,293<br>293	291<br>287<br>-	287<br>295<br>-	-<br>-<br>289,294	Yes<br>Yes<br>No*	287<br>286, 287, 291<br>286	-<br>-<br>-	-<br>-<br>-
α11-β15<br>**interaction with ATP-Mg^{2+}*	296-303<br>301-303	296<br>-<br>-	-<br>-<br>296	-<br>-<br>-	-<br>-<br>-	-<br>-<br>-	No*<br>No*<br>No*	296<br>-<br>-	-<br>-<br>-	-<br>-<br>-
CTD										
β15	304-309	-<br>309	305,307<br>308	309<br>-	-<br>-	-<br>-	Yes<br>No*	305, 306, 308<br>305	309<br>-	-<br>-

		309	305	-	-	-	No*	-	-	-
β15-β16	310-311	311<br>311<br>-	-<br>-<br>-	-<br>-<br>-	-<br>-<br>-	-<br>-<br>-	No*<br>No*<br>No*	-<br>-<br>-	-<br>-<br>-	-<br>-<br>-
β16	312-316	-<br>-<br>-	-<br>315,316<br>315	-<br>-<br>312	-<br>-<br>-	312,314,315<br>312<br>314,316	No<br>No<br>Yes	313<br>313, 316<br>316	-<br>-<br>-	-<br>-<br>-
β16-α12<br>**interaction with ATP-Mg^{2+}*<br>**interaction with inhibitor* ***1***	317-322<br>317,319,<br>320<br>319,321	-<br>-<br>-	-<br>317<br>317	-<br>-<br>-	-<br>-<br>-	317<br>-<br>-	No*<br>No*<br>No*	317<br>317<br>-	-<br>-<br>-	-<br>-<br>-
α12<br>**interaction with ATP-Mg^{2+}*	323-330<br>324,325,<br>328	-<br>-<br>-	-<br>-<br>-	-<br>324<br>-	-<br>-<br>-	325<br>-<br>-	No*<br>Yes*<br>-**	323, 324, 325<br>324<br>-	325<br>324<br>-	-<br>-<br>-
α12-β17	331-338	332,335-337<br>332,333,335-337<br>332,335,337	-<br>331<br>-	-<br>-<br>-	-<br>-<br>333	-<br>-<br>338	No*<br>No<br>No	331, 332, 333, 335, 337,338<br>331^{V}, 332, 335, 338<br>-	332<br>-<br>-	-<br>-<br>-
β17	339-344	341<br>-<br>-	340<br>339-341<br>341-343	-<br>-<br>-	-<br>-<br>-	342<br>342<br>340	No<br>No<br>No	339, 342, 343, 344<br>339<br>339, 342^{V}, 344	342<br>-<br>-	-<br>-<br>-
β17-α13<br>**interaction with inhibitor* ***1***	345-352<br>348	-<br>349,351<br>349	-<br>-<br>348	348<br>348<br>-	347<br>-<br>-	345,346<br>-<br>346	Yes<br>Yes<br>No	345, 347, 348, 349, 351<br>345, 348, 349<br>345, 348^{V}, 351^{V}	348<br>348<br>-	-<br>-<br>-
α13	353-358	-<br>-<br>-	-<br>356<br>353	353,356<br>-<br>356,358	358<br>-<br>-	-<br>-<br>-	Yes<br>No*<br>Yes*	353, 356<br>-<br>353	356<br>-<br>356,358	-<br>-<br>-
α13-β18	359-363	360,363<br>359-361,363<br>359-361,363	-<br>-<br>-	-<br>-<br>-	359<br>-<br>-	-<br>-<br>-	No<br>No<br>No	359, 361<br>-<br>-	363<br>-<br>361	-<br>-<br>-
β18	364-369	-<br>-<br>-	368<br>366-369<br>367	369<br>-<br>-	-<br>-<br>-	365<br>365<br>-	Yes<br>No<br>No	366<br>366<br>-	369<br>-<br>369	-<br>-<br>369
β18-α14	370-372	-<br>-<br>-	370<br>371,372<br>371,372	371,372<br>-<br>-	-<br>-<br>-	-<br>-<br>-	Yes<br>No<br>No	370, 372<br>370<br>-	-<br>-<br>372	-<br>-<br>372
α14	373-377	375<br>-<br>375	-<br>376,377<br>374,377	373,374<br>-<br>-	-<br>375<br>-	-<br>373,374<br>-	Yes<br>No<br>No	374, 377<br>-<br>-	-<br>-<br>-	-<br>-<br>-
α14-β19	378-383	382<br>-<br>-	-<br>378<br>379	379,383<br>379,382<br>-	-<br>-<br>382	-<br>-<br>380	Yes<br>Yes<br>No	380, 382<br>378, 379<br>-	379,383<br>379,382<br>-	-<br>-<br>-
β19	384-386	-<br>-<br>-	-<br>-<br>-	-<br>-<br>-	-<br>-<br>-	-<br>384<br>-	No<br>No<br>No	385, 386<br>384<br>386	-<br>384<br>-	-<br>-<br>-

β19-α15	387-390	-<br>-<br>389	388,389<br>387,390<br>387,390	390<br>-<br>-	-<br>-<br>-	-<br>-<br>-	Yes<br>No<br>No	387, 388, 389, 390<br>-<br>-	389,390<br>-<br>-	-<br>-<br>-
α15	391-401	-<br>-<br>-	391,394,397,398,<br>400<br>391,392,<br>394-396,400<br>391-397,400	395,401<br>397<br>-	-<br>-<br>-	-<br>393,398<br>-	Yes<br>Yes<br>No	400, 401<br>-<br>-	395,401<br>393,397<br>397	-<br>-<br>397
α15-β20	402-405	-<br>-<br>-	-<br>-<br>-	405<br>-<br>-	-<br>-<br>-	-<br>-<br>405	Yes<br>No<br>No	-<br>402<br>-	-<br>-<br>-	-<br>-<br>-
β20	406-409	-<br>-<br>-	406,409<br>406,408<br>408,409	408<br>-<br>-	-<br>-<br>-	407<br>409<br>-	Yes<br>No<br>No	406, 407<br>406, 407<br>-	408<br>-<br>-	-<br>-<br>-
β20-α16<br>**interaction with inhibitor **1***	410-421<br>414-416,<br>420,421	412,414<br>412<br>412	-<br>410,421<br>410,413	413,417<br>417<br>417	-<br>-<br>-	-<br>-<br>-	Yes*<br>Yes<br>Yes*	410,412,413,414,<br>421<br>410,414,421<br>421	412,413,417<br>417,421<br>417	-<br>417,421<br>-
α16<br>**interaction with inhibitor **1***	422-437<br>422,423	428,434<br>430,434<br>434	436<br>429,431,436<br>429,436	426,433<br>426<br>-	-<br>-<br>426	429,432<br>428,432<br>-	Yes<br>Yes<br>No*	429, 430<br>430<br>431^{V}	426, 433,434<br>426<br>431	-<br>426<br>-

Some deviation from this description for the $MurD_{inh}$ is explained in the text of the manuscript.

,,* At least half of the residues within the segment are not available from the assignment.

,,** No residues within the segment are available from the assignment.

^{V}Decay is observed in the CPMG plots, but the fitting results are less reliable.

Table S4: Number of segments with residues showing the dynamics of a particular group per domain for all three states of MurD. The number of segments referring to the ATP binding sites is given after the semicolon and separated from the number of binding sites of inhibitor **1** by a slash.

	**NTD (19 segments; ATP: 0 / inhibitor 1: 6)**						**CeD (32 segments; ATP: 6 / inhibitor 1: 5)**						**CTD (21 segments; ATP: 2 / inhibitor 1: 4)**					
	µs-ms ex.	G1	G2	G3	G4	G5	µs-ms ex.	G1	G2	G3	G4	G5	µs-ms ex.	G1	G2	G3	G4	G5
**$MurD_{apo}$**	4; 0/3	5; 0/2	8; 0/1	6; 0/2	5; 0/1	3; 0/2	20; 3/3	13; 3/3	21; 5/4	13; 2/1	5; 0/0	9; 3/1	19; 2/4	8; 0/2	8; 0/1	13; 0/3	3; 0/1	8; 2/3
**$MurD_{ACP}$**	2; 0/1	5; 0/3	13; 0/4	5; 0/2	5; 0/2	3; 0/1	20; 5/4	12; 1/2	23; 4/5	16; 3/3	4; 1/1	12; 4/1	15; 2/4	7; 0/3	14; 1/3	6; 1/3	1; 0/0	8; 0/1
**$MurD_{inh}$**	4; 0/2	6; 0/1	14; 0/4	2; 0/2	2; 0/0	5; 0/5	12; 2/4	9; 1/0	22; 5/4	6; 1/0	3; 0/0	15; 2/1	6; 0/2	7; 0/3	15; 1/4	3; 0/1	3; 0/1	6; 0/1

Table S5: Overview of the segments and the residues exhibiting conformational exchange with k_{ex} values for $MurD_{apo}$, $MurD_{ACP}$, and $MurD_{inh}$ labeled in black, purple, and green, respectively. The secondary structure annotations are taken from Bertrand *et al.*[1].

Segment = Secondary structure element	*Sequence*	*Conformational exchange determined by CPMG RD method and fitted to the 2-site CPMG model as derived and provided in[8]*					
		**$MurD_{apo}$**		**$MurD_{ACP}$**		**$MurD_{inh}$**	
		**Residue**	**k_{ex} (s^{-1})**	**Residue**	**k_{ex} (s^{-1})**	**Residue**	**k_{ex} (s^{-1})**
NTD							
β1-α1<br>*interaction with inhibitor **1**	12-14<br>12					12	825
*interaction with inhibitor **1**	32-35<br>34,35	34	13			34	0.001
β2-α2<br>*interaction with inhibitor **1**	36-40<br>36,37	37<br>39	2124<br>16238				
α2	41-45	42	5740	43	11730	43<br>44	926<br>4801
β4-α4<br>*interaction with inhibitor **1**	71-78<br>71-74	77	1523	73	7502		
α4	79-86					85	3694
CeD							
α5	94-101					94[V]<br>101	18633<br>21061
β6-α6<br>*interaction with ATP-Mg^{2+}	111-114<br>111-114			111<br>112	111<br>1682		
α6<br>*interaction with ATP-Mg^{2+}<br>*interaction with inhibitor **1**	115-128<br>115-117<br>115			121<br>126[V]	555<br>11094		
α6-β7	129-132			130	552		
β7	133-137	133<br>136	1348<br>233	136	743	135	420
β7-α7<br>*interaction with inhibitor **1**	138-142<br>138,139	139<br>140<br>141	5595<br>988<br>404	139<br>140<br>141	1407<br>86<br>2466		
α7	143-146			144[V]	1824		
α7-β8	147-152	147	4179				
β8<br>*interaction with ATP-Mg^{2+}<br>*interaction with inhibitor **1**	153-157<br>157	157	523	157	6		
β8-α8<br>*interaction with inhibitor **1**	158-159<br>159	158	730	158<br>159	944<br>598		
α8<br>*interaction with inhibitor **1**	160-164<br>161					163	1045
α8-β9	165-172	166<br>170	575<br>0.6	167[V]	11788		
loop β9-α1*-α9<br>*interaction with ATP-Mg^{2+}<br>*interaction with inhibitor **1**	177-189<br>178<br>182,183,186	181<br>182<br>184	1570<br>893<br>1890	179[V]<br>189	2709<br>1040	189	17301
α9	190-202	193		193<br>198	2431<br>13346	190<br>202	25<br>6
α9-β10	203-206	205	388				
β10-α2*-β11<br>**unresolved in X-ray structures**	212-227<br>(unresolved 221-225)	220	546	226	480	222<br>227[V]	1839<br>31
β11	228-230	228	12448	228	8262		
β11-β12	231-236	231<br>234	419<br>1971	233<br>236	1224<br>267		
β12	237-240	237<br>238<br>240	1175<br>3318<br>8360	238<br>240	5005<br>12698	240	4869
β12-β13<br>**unresolved in some X-ray structures**	241-246					244[V]	254
β13	247-250	249<br>250	2286<br>12995				
β13-β14	251-252	251	3653				
β14	253-257	255	1281	255	3302	253<br>257	1301<br>70
β14-α10	258-265	263	2348	258	6020	264	1440

		264	19				
α10<br>*interaction with ATP-Mg^{2+}	266-282<br>267,268,270,271			269	9487	272[V]<br>281[V]<br>282[V]	1<br>16568<br>8014
α10-α11	283-285			283[V]	8528		
α11	286-295	287	505	286<br>287<br>291	1822<br>854<br>2769	286	14039
α11-β15<br>*interaction with ATP-Mg^{2+}	296-303<br>301-303	296	1074				
CTD							
β15	304-309	305<br>306<br>308	1095<br>19<br>1308	305	614		
β16	312-316	313	506	313<br>316	289<br>1	316	2099
β16-α12<br>*interaction with ATP-Mg^{2+}<br>*interaction with inhibitor **1**	317-322<br>317,319,320<br>319,321	317	183	317	1634		
α12<br>*interaction with ATP-Mg^{2+}	323-330<br>324,325,328	323<br>324<br>325	554<br>1518<br>769	324	1217		
α12-β17	331-338	331<br>332<br>333<br>335<br>337<br>338	36<br>9584<br>561<br>591<br>1386<br>198	331[V]<br>332<br>335<br>338	494<br>825<br>1425<br>533		
β17	339-344	339<br>342<br>343<br>344	668<br>0.02<br>1417<br>179	339	957	339<br>342[V]<br>344	1405<br>19322<br>0.7
β17-α13<br>*interaction with inhibitor **1**	345-352<br>348	345<br>347<br>348<br>349<br>351	1736<br>1999<br>54<br>2842<br>920	345<br>348<br>349	8509<br>1397<br>1533	345<br>348[V]<br>351[V]	141<br>5959<br>1048
α13	353-358	353<br>356	20<br>140			353	10
α13-β18	359-363	359<br>361	14613<br>781				
β18	364-369	366	64	366	2629		
β18-α14	370-372	370<br>372	487<br>729	370	1593		
α14	373-377	374<br>377	9<br>13592				
α14-β19	378-383	380<br>382	35<br>494	378<br>379	28<br>831		
β19	384-386	385<br>386	502<br>361	384	75	386	250
β19-α15	387-390	387<br>388<br>389<br>390	734<br>231<br>1046<br>34				
α15	391-401	400<br>401	4191<br>644				
α15-β20	402-405			402	405		
β20	406-409	406<br>407	561<br>375	406<br>407	234<br>383		
β20-α16<br>*interaction with inhibitor **1**	410-421<br>414-416,<br>420,421	410<br>412<br>413<br>414<br>421	11<br>3584<br>2714<br>700<br>17805	410<br>414<br>421	232<br>7379<br>7	421	18113
α16<br>*interaction with inhibitor **1**	422-437<br>422,423	429<br>430	236<br>269	430	571	431[V]	18317

[V] Decay is visible in the CPMG plots, but less pronounced.

Table S6: Comparison of prediction of conformational exchange dynamics on μs-ms time scale using PCA of reduced spectral densities, increased values of R_1R_2 products and R_{ex} calculated using approach of Kneller *et al.*[7] with observed conformational exchange dynamics using CPMG-RD NMR method.

	**MurD$_{apo}$**	**MurD$_{ACP}$**	**MurD$_{inh}$**
**Agreement between data**	*Segments (%)*	*Segments (%)*	*Segments (%)*
PCA vs CPMG	51 (71%)	46 (64%)	52 (72%)
R_1R_2 vs CPMG	48 (67%)	41 (57%)	42 (58%)
Kneller vs CPMG	26 (36%)	37 (51%)	44 (61%)
**Observed CPMG**	In total 43	In total 36	In total 22
PCA prediction	26 (61%)	19 (53%)	7 (32%)
R_1R_2 prediction	26 (61%)	16 (44%)	8 (36%)
Kneller prediction	2 (5%)	6 (17%)	2 (9%)

Supplementary References

1. Bertrand, J. A. *et al.* 'Open' structures of MurD: Domain movements and structural similarities with folylpolyglutamate synthetase. *J Mol Biol* **301**, (2000).
2. Farrow, N. A. *et al.* Backbone Dynamics of a Free and a Phosphopeptide-Complexed Src Homology 2 Domain Studied by 15N NMR Relaxation. *Biochemistry* **33**, 5984–6003 (1994).
3. Seibold, S., Lovell, S., Liu, L. & Battaile, K. P. Crystal Structure of Apo UDP-N-acetylmuramoylalanine--D-glutamate ligase (MurD) from E. coli (ATP bound). *PDB: 8VW2* (2024).
4. Sosič, I. *et al.* Second-generation sulfonamide inhibitors of d-glutamic acid-adding enzyme: Activity optimisation with conformationally rigid analogues of d-glutamic acid. *Eur J Med Chem* **46**, (2011).
5. Šink, R. *et al.* Crystallographic Study of Peptidoglycan Biosynthesis Enzyme MurD: Domain Movement Revisited. *PLoS One* **11**, e0152075 (2016).
6. Bertrand, J. A. *et al.* Determination of the MurD mechanism through crystallographic analysis of enzyme complexes. *J Mol Biol* **289**, (1999).
7. Kneller, J. M., Lu, M. & Bracken, C. An effective method for the discrimination of motional anisotropy and chemical exchange. *J Am Chem Soc* **124**, (2002).
8. Korzhnev, D. M., Kloiber, K. & Kay, L. E. Multiple-quantum relaxation dispersion NMR spectroscopy probing millisecond time-scale dynamics in proteins: Theory and application. *J Am Chem Soc* **126**, 7320–7329 (2004).
