# Supplementary material for: Mechanistic insight into the dynamics of Mur ligase through a comprehensive timescale-specific approach
Source: Commun Chem. 2025 Sep 29;8:285. doi: 10.1038/s42004-025-01675-z (PMC12480691; doi:10.1038/s42004-025-01675-z)
Supplement: Supplementary file 2 — Description of Additional Supplementary Files [file 42004_2025_1675_MOESM2_ESM.pdf]

Description of Additional Supplementary Files

File name: Supplementary Data 1

Description: 1HN, 15N chemical shifts of the MurD apo and bound states to support the NMR spectra in Figure 1b and Figure 6b.

File name: Supplementary Data 2

Description: Numerical data to support the graphical representation of Figure 2, insets a, c and d.

File name: Supplementary Data 3

Description: Numerical data to support the graphical representation of Figure 3, insets c, d and e.

File name: Supplementary Data 4

Description: Numerical data to support the graphical representation of Figure 5, insets i), ii) and iii).

File name: Supplementary Data 5

Description: Coordinate file of the initial frame of MurDapo.

File name: Supplementary Data 6

Description: Coordinate file of the final frame of MurDapo.

File name: Supplementary Data 7

Description: Coordinate file of the initial frame of MurDATP.

File name: Supplementary Data 8

Description: Coordinate file of the final frame of MurDATP.

File name: Supplementary Data 9

Description: Coordinate file of the initial frame of MurDinh.

File name: Supplementary Data 10

Description: Coordinate file of the final frame of MurDinh.
